# Enzyme/Nanocopper Hybrid Nanozymes: Modulating Enzyme-like Activity by the Protein Structure for Biosensing and Tumor Catalytic Therapy

**DOI:** 10.1021/acsami.0c20501

**Published:** 2021-01-21

**Authors:** Noelia Losada-Garcia, Ana Jimenez-Alesanco, Adrian Velazquez-Campoy, Olga Abian, Jose M. Palomo

**Affiliations:** † Department of Biocatalysis, Institute of Catalysis (CSIC), c/Marie curie 2, Cantoblanco Campus UAM, 28049 Madrid, Spain; ‡ Fundación ARAID, Gobierno de Aragón, 50018 Zaragoza, Spain; § Instituto de Biocomputación y Física de Sistemas Complejos, Joint Units IQFR-CSIC-BIFI, and GBsC-CSIC-BIFI, Universidad de Zaragoza, 50009 Zaragoza, Spain; ∥ Fundación Instituto de Investigación Sanitaria de Aragón (IIS Aragón), 50009 Zaragoza, Spain; ⊥ Centro de Investigación Biomédica en Red en el Área Temática de Enfermedades Hepáticas y Digestivas (CIBERehd), 28029 Madrid, Spain; # Departamento de Bioquímica y Biología Molecular y Celular, Universidad de Zaragoza, 50009 Zaragoza, Spain; ∇ Instituto Aragonés de Ciencias de la Salud (IACS), 50009 Zaragoza, Spain

**Keywords:** nanozymes, copper hybrids, nanoparticles, oxidase-like activity, biosensors, cytotoxicity

## Abstract

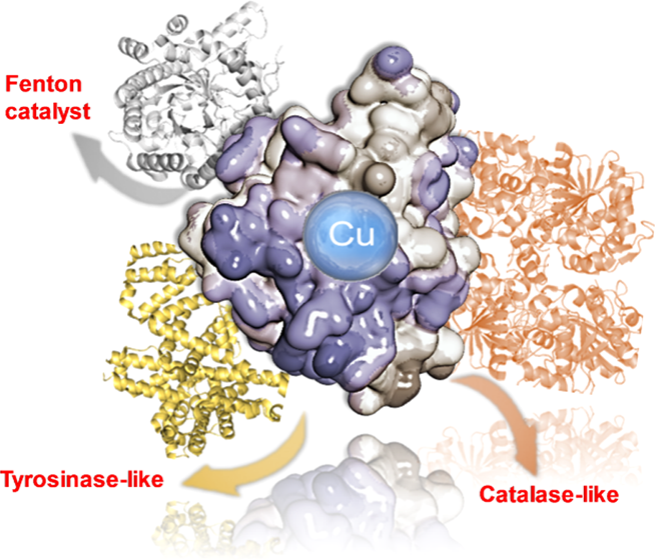

Artificial enzymes with modulated enzyme-mimicking activities of natural systems represent a challenge in catalytic applications. Here, we show the creation of artificial Cu metalloenzymes based on the generation of Cu nanoparticles in an enzyme matrix. Different enzymes were used, and the structural differences between the enzymes especially influenced the controlled the size of the nanoparticles and the environment that surrounds them. Herein, we demonstrated that the oxidase-like catalytic activity of these copper nanozymes was rationally modulated by enzyme used as a scaffold, with a special role in the nanoparticle size and their environment. In this sense, these nanocopper hybrids have confirmed the ability to mimic a unique enzymatic activity completely different from the natural activity of the enzyme used as a scaffold, such as tyrosinase-like activity or as Fenton catalyst, which has extremely higher stability than natural mushroom tyrosinase. More interestingly, the oxidoreductase-like activity of nanocopper hybrids was cooperatively modulated with the synergistic effect between the enzyme and the nanoparticles improving the catalase activity (no peroxidase activity). Additionally, a novel dual (metallic and enzymatic activity) of the nanozyme made the highly improved catechol-like activity interesting for the design of 3,4-dihydroxy-l-phenylalanine (l-DOPA) biosensor for detection of tyrosinase. These hybrids also showed cytotoxic activity against different tumor cells, interesting in biocatalytic tumor therapy.

## 1 Introduction

One of the key advantages of enzymes is the high selectivity and activity against a particular reaction; however, outside of cells, they present low activity against non-natural substrate and low stability in different media than biological environment, which are important drawbacks for commercial applications. Also, difficult and time-consuming purification steps, which resulted in a final high cost of the product, limit their industrial application.

Nanozymes have emerged in the last years as one of the most interesting alternatives to natural enzymes, and even conventional enzyme mimics, as artificial biocatalytic tools for decontamination, biosensor, and biomedical applications.^1−14^

At this point, nanozymes show unique advantages over natural enzymes offering robustness to harsh environments, high stability, long-term storage, ease of modification, and lower manufacturing cost than protein enzymes. Additionally, nanozymes possess inherent nanomaterial properties, providing not only a simple substitute of enzymes but also a multimodal platform interfacing complex biologic environments.^3,15,16^

However, one of the most challenging tasks is the development of novel strategies to synthesize nanozymes that mimic a particular natural activity, especially capable of specific enzymatic activity that has not been studied much.

Most of the currently developed nanozymes still face several challenges such as limited specificities and catalytic activities compared with their natural counterparts.^17,18^ To overcome these challenges, several strategies based on the functionalization of nanozymes surface or designing novel nanozymes with structures similar to the active site of natural enzymes have been described.^19,20^

Thus, creating new properties with respect to natural enzymes with improved stability or even finding synergistic processes between enzyme and metallic catalytic centers^21−23^ are challenging.

In particular, mimics of copper metalloenzymes is an important case, considering the essential biological role of these enzymes.^24−28^ Phenol oxidases (catechol oxidases, tyrosinases (TYR)), catalases, and superoxide dismutase activities are involved in many different cellular process, and deficiency or malfunction of these activities is postulated to be related to the pathogenesis of many age-associated degenerative diseases like diabetes mellitus, hypertension, anemia, vitiligo, Alzheimer’s disease, Parkinson’s disease, bipolar disorder, cancer, and schizophrenia.^29−33^

In this work, we demonstrate how to modulate the particular enzyme-like activity of novel copper nanohybrids, formed by copper nanoparticles (as active sites) created in a protein environment (as a scaffold), where precisely the used enzyme plays a fundamental role.

**Figure 1:**
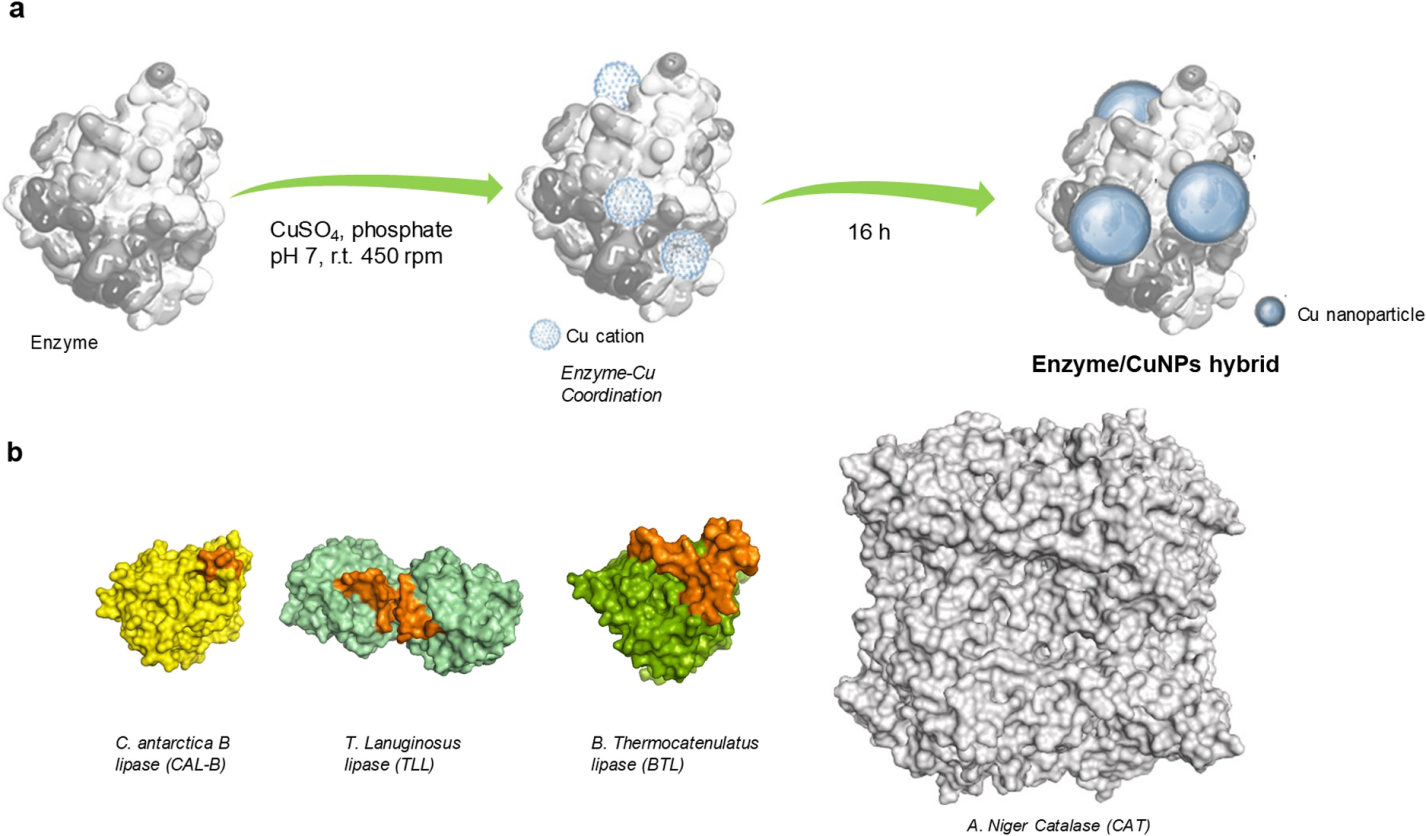
General concept and design of enzyme/CuNP hybrids as novel nanozymes. (a) Synthetic strategy. (b) Different enzymes used as protein scaffolds (the orange color represents the oligopeptide lid in lipases).

Here, we found that depending on the enzyme used in the synthesized hybrid (Figure 1), it was possible to obtain Cu nanozymes with modulated activity by altering the nanoparticle morphology and reactivity. For that purpose, enzymes with different natures, behaviors, and sizes were tested, with the ability to mimic particular enzyme activity completely different from the natural activity of the enzyme used as a scaffold, the highest tyrosinase-like activity, or even other oxidase activity in Fenton processes,^34^ for catalysis, for example, applied in biocatalytic tumor therapy.^35,36^

Furthermore, a synergistic effect between the enzyme as a scaffold and the CuNPs was observed for an enhanced catalase activity.

## 2 Experimental Section

### 2.1 Chemicals

Lipase B from *Candida antarctica* (CALB) solution (Lipozyme CALB), lipase from *Thermomyces lanuginosus* (TLL) solution (Lipozyme TL 100L), catalase from *Aspergillus niger* (CAT) solution (Catazyme), and glucose oxidase (Gluzyme Mono 10.000 BG) (GOX) were purchased from Novozymes (Copenhagen, Denmark). Genetically modified lipase from (*(Geo)Bacillus thermocatenulatus* without native cysteines (C65S/C296S) BTL) was produced by Dr. de las Rivas and purified following the previous report (obtaining a solution of 2.5 mg lipase/mL by Bradford assay determination).^37^ Copper(II) sulfate pentahydrate [Cu_2_SO_4_·5H_2_O] and hydrogen peroxide (33%) were from Panreac (Barcelona, Spain). *p*-Aminophenol (*p*NP), sodium phosphate, sodium acetate, tyrosinase (TYR) from mushroom, 2,2′-azino-bis(3-ethylbenzothiazoline-6-sulfonic acid)diammonium salt (ABTS), benzoquinone, and hydroquinone were purchased from Sigma-Aldrich (St. Louis, MO). 3,4-Dihydroxy-l-phenylalanine (l-DOPA) was from Alfa-Aesar (MA, EEUU). 3,4-Dihydroxy-l-phenylalanine methyl ester hydrochloride (l-DOPA methyl ester (DOPAME)) was purchased from Carbosynth (Berkshire, U.K.). Horseradish peroxidase (HRP) was from Thermo Scientific (Madrid, Spain).

### 2.2 Instrumentation

Cu nanoparticles sizes and morphology were determined by transmission electron microscopy (TEM) and high-resolution TEM (HRTEM). Images were obtained on a 2100F microscope (JEOL, Tokyo, Japan) equipped with an energy-dispersive X-ray (EDX) detector INCA X-sight (Oxford Instruments, Abingdon, U.K.). Interplanar spacing in the nanostructures was calculated using inversed Fourier transform spectroscopy with the GATAN digital micrograph program (Corporate Headquarters, Pleasanton, CA). Scanning electron microscopy (SEM) imaging was performed on a TM-1000 microscope (Hitachi, Tokyo, Japan). Inductively coupled plasma-optical emission spectrometry (ICP-OES) was performed on an OPTIMA 2100 DV instrument (PerkinElmer, Waltham, MA). X-ray diffraction (XRD) patterns were obtained using a Texture Analysis D8 Advance diffractometer (Bruker, Billerica, MA) with Cu Kα radiation. To recover the nanobiohybrids, a Biocen 22 R (Orto-Alresa, Ajalvir, Spain) refrigerated centrifuge was used. Spectrophotometric analyses were run on a V-730 spectrophotometer (JASCO, Tokyo, Japan). A synergy HT (BioTek) plate reader was used for cell viability assays.

### 2.3 General Synthesis of Enzyme–Cu(II)NP Hybrids

A corresponding amount of enzyme was added to 60 mL of sodium phosphate buffer (0.1 M, pH 7) to finally achieve an enzyme concentration of 0.27–0.3 mg/mL. In the case of CALB solution, 1.8 mL (9 mg lipase/mL determined by Bradford assay) was added; 0.75 mL of TLL solution (24 mg/mL determined by Bradford assay) and 0.5 mL of CAT (32 mg/mL determined by Bradford assay) were added, respectively. In the case of BTL, 20 mL (0.4 mg/mL solution containing 0.5% (w/v) of Triton X-100 from the purification step) was added, corresponding to an enzyme concentration of 0.13 mg/mL. The corresponding enzyme solution was poured in a 100 mL glass bottle containing a small magnetic bar stirrer (12 × 4.5 mm^2^). The solution was stirred in a magnetic agitator at 380–450 rpm for 1–2 min. Then, 600 mg of Cu_2_SO_4_·5H_2_O (10 mg/mL) was added to the protein solution and it was maintained for 16 h at room temperature (rt). After that, in all cases, the mixture was centrifuged at 8000 rpm for 5 min (10 mL per falcon-type tube). The generated pellet was resuspended in 15 mL of water, washed and centrifuged again at 8000 rpm for 5 min, and the supernatant was removed. The process was repeated twice more. Finally, the supernatant was removed and the pellet of each falcon was resuspended in 2 mL of water; each solution collected in a cryotube was frozen with liquid nitrogen and lyophilized for 16 h. After that, in all cases, approximately 350 mg of a blue solid was obtained. The different hybrids were called as **Cu-CALB**, **Cu-TLL**, **Cu-CAT**, and **Cu-BTL**. In the case of CAT, the protocol was repeated avoiding the lyophilization step, conserving the catalyst as a blue liquid suspension. This was called **Cu-CAT-NL**. Characterization of the different Cu hybrids was performed by XRD, SEM, TEM, HRTEM, circular dichroism (CD), and fluorescence analysis.

### 2.4 Circular Dichroism Measurements

Circular dichroism (CD) spectra of the different lipases were recorded in a Chirascan spectropolarimeter (Applied Photophysics) at 25(±1) °C. Far-UV spectra were recorded at wavelengths between 190 and 260 nm in a 0.1 cm path-length cuvette. Near-UV spectra were recorded at wavelengths between 250 and 310 nm in a 1 cm path-length cuvette. Protein concentrations were 20 and 10 μM, respectively, in phosphate-buffered saline (PBS), pH 7.2 (bioMerieux).

### 2.5 Fluorescence Spectroscopy Measurements

Fluorescence measurements were performed in a Varian Cary Eclipse fluorescence spectrophotometer (Agilent Technologies) monitoring the intrinsic tryptophan fluorescence in 2 μM of hybrid solutions, using an excitation wavelength of 280 nm, with excitation and emission bandwidths of 5 nm and recording fluorescence emission spectra between 300 and 400 nm with 1 nm step. All spectroscopic measurements were made in water.

### 2.6 Tyrosinase-like Activity Assay

3,4-Dihydroxy-l-phenylalanine (l-DOPA) (4 mg, 1 mM) or l-DOPA methyl ester (5 mg, 1 mM) was added to a 20 mL water solution, 0.1 M buffer sodium phosphate (pH 7), or 0.1 M buffer sodium acetate (pH 4). To initialize the reaction, 5 mg of Cu–enzyme hybrid or 50 μL of commercial mushroom tyrosinase (TYR) (1 mg/mL solution in distilled water) was added to 2 mL of DOPA solution and the mixture was slight stirred (roller) at room temperature. In the case of solid Cu hybrids, at different times, the mixture was centrifuged at 3000 rpm for 1 min and the absorbance of the supernatant (at different times) was measured at 475 nm in a JASCO V-730 UV spectrophotometer. Then, the Abs/min was calculated with these values in each case. In the case of tyrosinase, the increase of absorbance was directly measured at 475 nm with a UV spectrophotometer using the kinetic program. An enzyme activity unit (U) was defined as the amount of enzyme causing an increase of absorbance by 0.001/min at 25 °C.^38^ Experiments were also conducted in the presence of different concentrations of H_2_O_2_ (0–50 mM).

### 2.7 Catalase-like Activity Assay

Hydrogen peroxide (H_2_O_2_) (33% (w/w)) solution in distilled water was prepared to obtain a final concentration of 50 mM. The solution pH was adjusted to 7 using NaOH. To start the reaction, 2 mg of the Cu hybrid or 100 μL of Catazyme 25L (31 mg/mL) was added to 2 or 10 mL of the 50 mM solution at room temperature, respectively. The reaction was followed by measuring the degradation of hydrogen peroxide recording the decrease of absorbance spectrophotometrically at 240 nm in quartz cuvettes of 1 cm path length, at different times. To determine the catalase activity for each catalyst, the ΔAbs/min value was calculated using the linear portion of the curve (ΔAbs_S_).

The specific activity (U/mg) was calculated using the following equation
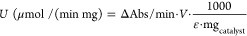
where the molar extinction coefficient (ε) used was 43.6 M^–1^ cm^–1^ and mg of enzyme or Cu content was used.

### 2.8 Fenton Catalyst Assay

*p*-Aminophenol (*p*AP) (1 mg) was dissolved in solutions (10 mL) of distilled water, and 100 mM hydrogen peroxide (1%, v/v) was added. To initialize the reaction, 10 mL of this solution was added to a glass bottle containing 3 mg of Cu hybrid and stirred gently at room temperature on an orbital shaker (320 rpm). At different times, samples (100 μL) were taken and the reaction was followed by high-performance liquid chromatography (HPLC). The samples were first centrifuged at 8000 rpm for 5 min and then 50 μL was diluted 20 times in bidistilled water before injection. HPLC column was C8 kromasil 150 × 4.6 mm^2^ AV-2059. HPLC conditions were: an isocratic mixture of 15% acetonitrile and 85% bidistilled water, UV detection at 270 nm, and a flow rate of 0.4 mL/min. Under these conditions, retention times of *p*AP and H_2_O_2_ were 8.5 and 4.2 min, respectively. The possible adsorption of substrate to the catalyst was first tested, and without the presence of hydrogen peroxide, no reaction was observed and the full area of the substrate was unaltered in the HPLC analysis.

In the case of **Cu-CALB**, the reaction was repeated in the presence of 0.1 mmol (2,2,6,6-tetramethylpiperidin-1-yl)oxyl (TEMPO) (48 mg of polymer-bound TEMPO).

### 2.9 Stabilization of the Cu Nanozymes

The stability of different enzyme/CuNP hybrids was evaluated by incubating them for 1 h at different temperatures in the presence of co-solvent or additives (1 mM of known tyrosinase inhibitors). Then, the tyrosinase-like activity of hybrids and enzymatic activity of tyrosinase (TYR) from mushroom was used for monitoring the stability, considering the activity at 25 °C in each case as 100%. The activity was determined using the DOPA assay described above. In the case of the presence of tyrosinase inhibitors, the activity evaluation was performed at 25 °C in aqueous media.

### 2.10 Cell Cultures

HT29 (human colon adenocarcinoma) and HeLa (human cervix epithelioid carcinoma) cells were obtained from ATCC and maintained in Dulbecco’s modified Eagle’s medium (DMEM) (PAN-Biotech GmbH, Germany) supplemented with 10% fetal bovine serum (FBS), 1% penicillin/streptomycin (P/S), and 1% non-essential amino acids (NEAAs) at 37 °C with 5% CO_2_.

### 2.11 Cell Viability Assays

Cellular cytotoxicity was assessed in two cell lines: HT29 and HeLa cells. Cells were plated in 96-well plates (8000 cells/90 μL/well in HeLa cells; 9000 cells/90 μL/well in HT29 cells) with supplemented DMEM without phenol red. After 24 h, 10 μL of several serial dilutions of the compounds was added to the cells (the solutions were prepared at 10× and the maximum concentration of compounds added to cells was the one in which there were 5% of H_2_O in each solution). The cells were in presence of the compounds during 24 h, and after this period, the cytotoxicity was checked by a 3-(4,5-dimethylthiazol-2-yl)-2,5-diphenyl tetrazolium bromide (MTT)-based assay. 3-(4,5-Dimethylthiazol-2-yl)-2,5-diphenyl tetrazolium bromide (MTT) reagent (Sigma Corp., St. Louis, MO) was prepared by dissolving 5 mg in 1 mL of PBS. The stock solution was protected from light and stored at 4 °C. To determine cytotoxicity, media was removed from wells and 50 μL of the working MTT solution (1 mg/mL in DMEM without phenol red) was added to each well and incubated at 37 °C for 3 h in a humidified, 5% CO_2_ atmosphere. After that, the media was carefully removed and the cells were solubilized into 100 μL of isopropanol (Scharlab, S.L., Barcelona, Spain). After 15 min shaking cautiously and protecting from light, the absorbance was recorded at 570 nm (reference wavelength: 650 nm) using a Synergy HT (BioTek) plate reader. Each experiment was performed in quadruplicate, repeated at least two times, and normalized regarding untreated cell viability.

## 3 Results and Discussion

### 3.1 Synthesis and Characterization of Enzyme/CuNP Hybrids

The preparation of copper nanoparticle hybrids as novel nanozymes was attempted. Here, different enzymes—which involved different conformational structures, dimeric or multimeric complexes, or even introducing post-translational modifications—were used in the preparation of enzyme/CuNP hybrids. In all cases, protein amounts of 0.27–0.3 mg/mL dissolved in phosphate buffer pH 7 were incubated with CuSO_4_ (10 mg/mL) for 16 h. A solid obtained after centrifugation indicates the final process of the Cu hybrid synthesis. The strategy was performed directly in aqueous solution at room temperature using the following enzymes: three different lipases, lipase from *C. antarctica* B (CALB) (33 kDa), lipase from *(G.)B. thermocatenulatus* (BTL) (43 kDa) and lipase from *T. lanuginosus* (TLL) (33 kDa, dimer), and a catalase produced by a genetically modified strain of the fungus *A. niger* (CAT) (80 kDa, tetramer).

The final step in obtaining enzyme/CuNP hybrids involved the lyophilization of a frozen suspension of the solid. At this term, we could obtain at multimilligram scale a set of different Cu hybrids called as **Cu-CALB**, **Cu-TLL**, **Cu-BTL**, or **Cu-CAT**.

**Figure 2:**
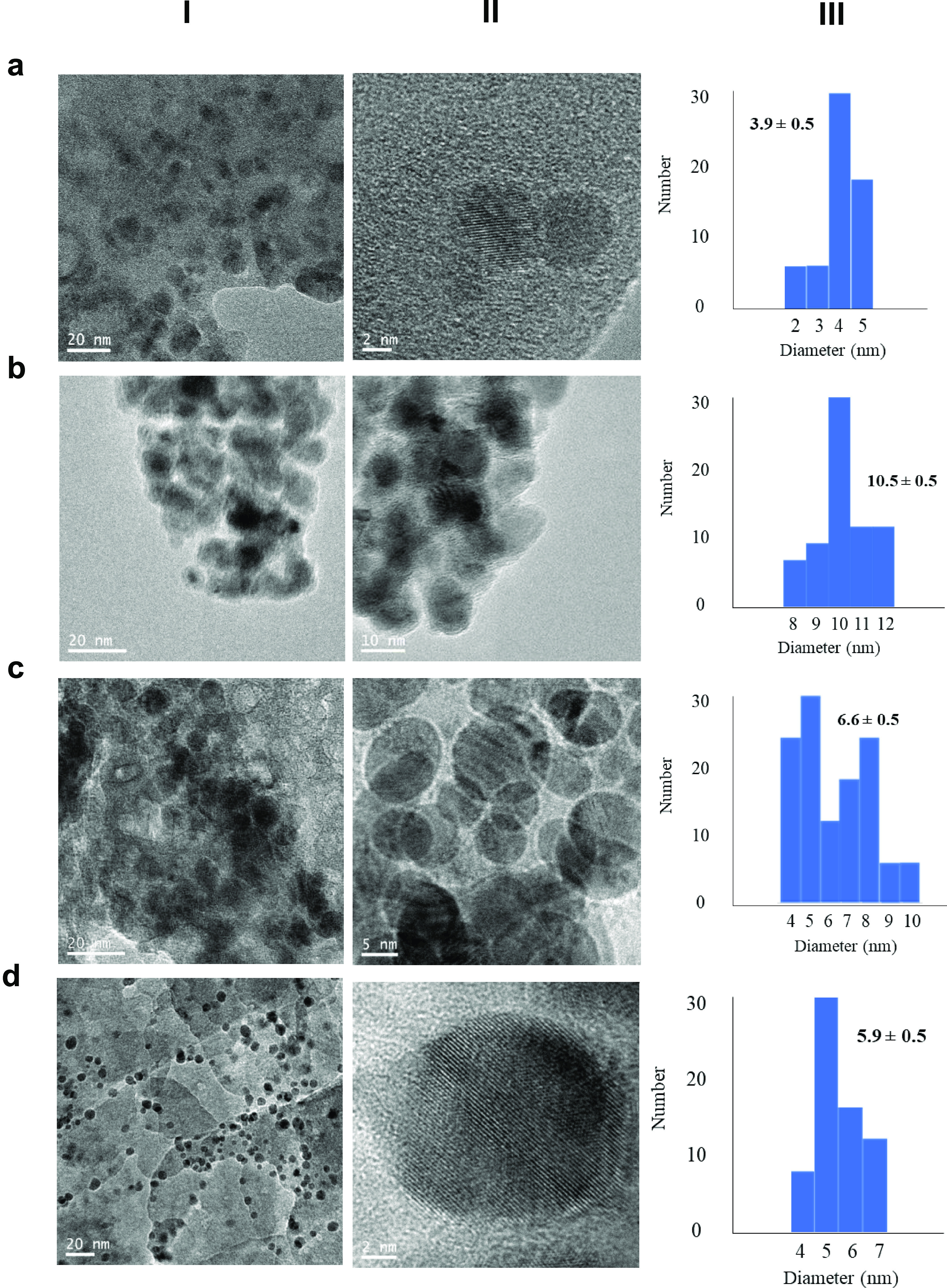
Characterization of different enzyme/CuNP hybrids: (a) **Cu-CALB**, (b) **Cu-TLL**, (c) **Cu-BTL**, and (d) **Cu-CAT**. (I) Transmission electronic microscopy (TEM) images; (II) high-resolution (HR)-TEM images; and (III) nanoparticle size distribution.

Wide-angle X-ray diffraction (XRD) analyses revealed a similar XRD pattern for all of enzyme/CuNP hybrids, displaying characteristic peaks of Cu_3_(PO_4_)_2_ (matched well with JCPDS card no. 00-022-0548 and some reports^39,40^) as unique copper species (Figure S1). Transmission electronic microscopy (TEM) revealed the formation of small-size crystalline nanoparticles on the protein network in the Cu hybrids (Figures 2 and S2–S5). However, the homogeneous nanoparticles distribution and especially the nanoparticles size were different depending on the enzyme used as a scaffold. Cu(II) nanoparticles around 3–10 nm were generated, and we could see in some cases their size increased with the size of the protein. Larger nanoparticles were obtained with TLL (10 nm) (Figure 2b). The explanation of the size and the less homogeneity of the nanoparticles with this enzyme could be because of the extremely high capacity of this enzyme to aggregation,^41^ which has been crystallized in tetrameric or even octameric form; therefore, we can consider this case a real enzyme unit as more than 100 kDa. Comparing CALB to CAT, we can clearly see that the size of nanoparticles increased from 3.9 (for CALB, Figure 2a) to around 6 nm because of the protein size (Figure 2).

Thus, in the case of BTL, although it is a slightly larger protein than CALB, Cu(II) nanoparticles of 6.6 nm were obtained (Figure 2c).

ICP-OES analysis showed that the copper content in the different hybrids was quite similar (34–37%), with the exception of TLL with a Cu content of 44% (Table S1).

### 3.2 Spectroscopic Characterization of the Enzyme/CuNP Hybrids

**Figure 3:**
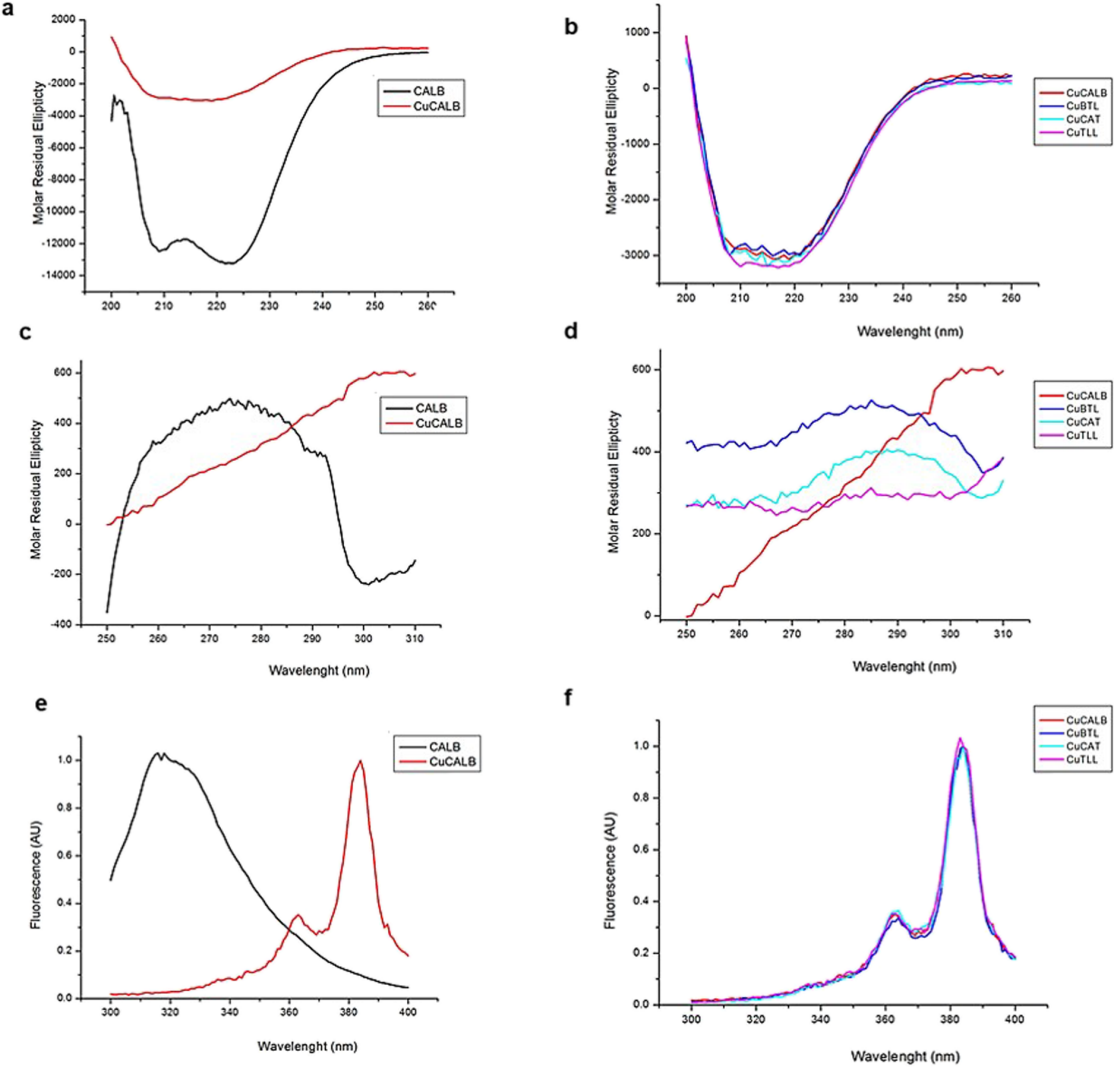
Spectroscopy characterization (CD and fluorescence) of the different enzyme/CuNP hybrids. (a) Far-CD spectra of **Cu-CALB** hybrid compared to soluble CALB. (b) Far-CD spectra of the different Cu hybrids. (c) Near-CD spectra of **Cu-CALB** hybrid compared to soluble CALB. (d) Near-CD spectra of the different Cu hybrids. (e) Fluorescence spectra of **Cu-CALB** hybrid compared to soluble CALB. (f) Fluorescence spectra of the different Cu hybrids. Fluorescence measurements were performed using an excitation wavelength of 280 nm, with excitation and emission bandwidths of 5 nm.

Far-circular dichroism (CD) for studying the effect of the modification on the secondary structure of enzymes, as well as near-CD and fluorescence assays were performed for analyzing the effect on the tertiary structure (Figure 3). The far-CD spectrum signal obtained was lower in **Cu-CALB** hybrids in comparison to soluble CALB, but there was still some residual α helix secondary structure (according to the peaks at 208 and 222 nm) (Figure 3a). The same behavior was found for the rest of enzyme/CuNP hybrids (Figure 3b). The shape and amount of residual signal were similar in all Cu hybrids.

CALB near-CD spectrum signal was low, and in case of enzyme/CuNP hybrids, it was lower (or even negligible in some cases) (Figure 3c,d) which means that the tertiary structure was altered upon Cu–enzyme derivative formation.

The fluorescence of protein tryptophan’s emission at 345 nm (upon 275 nm excitation) has been demonstrated to be quenched by copper and shifted to higher wavelengths (around 400 nm).^42^ A similar behavior was found in these enzyme/CuNP hybrids. Tryptophan fluorescence signal of CALB-soluble enzyme exhibited a peak at 320 nm (upon 280 nm excitation), which was not present in **Cu-CALB** hybrids (Figure 3e) neither in the rest of enzyme/CuNP hybrids (Figure 3f). However, we observed a clear signal at 385 nm in the fluorescence spectra of all six Cu hybrids (Figure 3f), which is characteristic of a Cu^2+^–enzyme complex formation.^43^

### 3.3 Tyrosinase-Mimicking Activity of Different Enzyme/CuNP Hybrids

**Figure 4:**
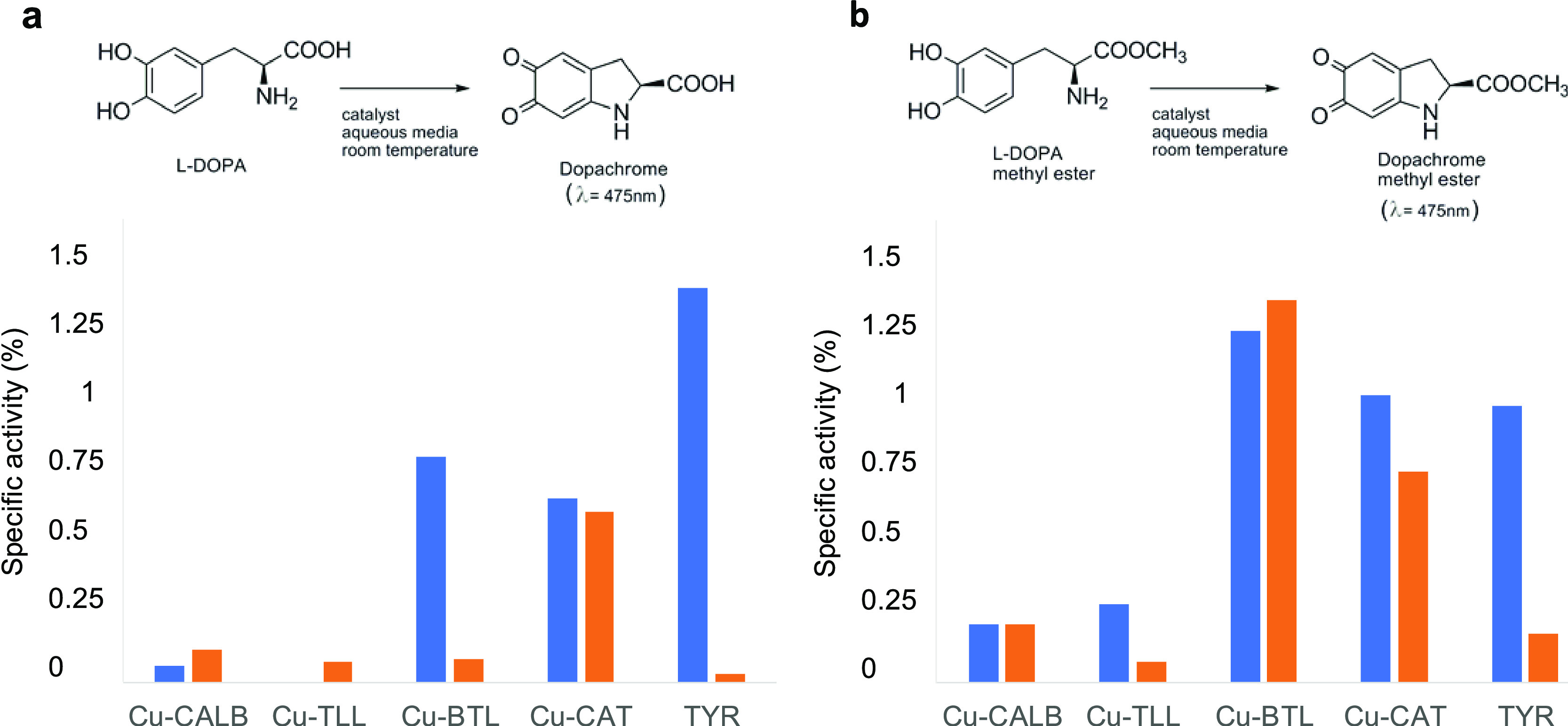
Tyrosinase-like activity of different enzyme/CuNP hybrids. (a) Oxidation of l-DOPA at different conditions expressed in values of specific activity (U/mg_cu_ for hybrids or mg_prot_ for TYR). (b) Oxidation of l-DOPA methyl ester at different conditions expressed in values of specific activity (U/mg). Distilled water (blue column), pH 4 (orange column). The activity value of TYR is ×10^2^.

Copper tyrosinases (Tyr) represent a very important class of oxidases with a key role in catalytic biological systems.^26,27^ However, high difficulty to obtain highly stable proteins with a high level of expression makes them an excellent example of enzyme type where artificial metalloenzyme can be a challenge. These enzymes can catalyze: (i) the o-hydroxylation of monophenols to *o*-diphenols as well as (ii) the oxidation of *o*-diphenols to produce *o*-quinones. In contrast, and by definition, catechol oxidase can only catalyze the oxidation of *o*-diphenols to their corresponding *o*-quinones. Here, catechol oxidase-like activity of the different enzyme/CuNP hybrids was evaluated using l-3,4-dihydroxyphenylalanine (DOPA) and its derivative l-DOPA methyl ester (DOPAME) as a substrate. Both of these compounds do not absorb in the visible region; however, the oxidation produces a chromogenic product (dopachrome), which is brown in color and absorbs at 475 nm (Figure 4).

In distilled water and using DOPA as substrate, **Cu-BTL** showed the highest catechol oxidase activity, almost 1 U/mg, between all of the enzyme/Cu hybrids (Figure 4a). However, using the other two Cu hybrids synthesized using lipases, little (**Cu-CALB**) or even no catechol-like activity (**Cu-TLL**) was observed.

However, the evaluation of the activity under acidic conditions (pH 4) shows a significant difference, especially for the hybrid synthesized using BTL, which exhibited 10 times less catechol-like activity, being at these conditions quite similar for all of the lipases. This result was also detected when the catechol activity of mushroom from *Agaricus bisporus* tyrosinase (TYR) was evaluated. This is a well-known enzyme that is normally used as a model of the human tyrosinase because both have a very high structural homology.^44^

However, when catalase was used as an enzyme for Cu hybrid formation, the **Cu-CAT** hybrid showed only a slightly lower activity than **Cu-BTL** at pH around 6–7, although it was stable to pH change, with hardly any variation in activity, being the most active Cu hybrid (more than 5 times than all others) at pH 4 (Figure 4a). This is interesting because under these conditions, the catechol activity of this **Cu-CAT** hybrid is only slightly lower than that of natural TYR (which showed a specific activity of 3 U/mg) (Figure 4a and Table S2).

In all hybrids, the catechol-like activity is provided exclusively by the CuNPs synthesized in the hybrids, and no catechol activity was found with enzymes used as a scaffold (Table S2).

The catechol-like activity of the Cu hybrids using DOPAME as a substrate demonstrated the important effect of blocking the negative charge of the carboxylic group in the substrate. In almost all cases, the activity of the hybrids increased using DOPAME with respect to the activity against DOPA. However, TYR showed a decrease (almost 2-fold in distilled water) in the enzymatic activity when the DOPA derivative was used (Figure 4b and Table S3). As occurred with l-DOPA, the different enzymes used in the synthesis of Cu hybrid did not show activity against DOPAME (Table S3).

In distilled water as a solvent, the **Cu-BTL** hybrid showed the highest activity between all hybrids (Figure 4b). **Cu-TLL** was active against DOPAME (0.275 U/mg), in similar values to **Cu-CALB**.

The effect of pH concerning the structure of the protein on activity was clearly shown when the hybrid activity was evaluated at pH 4 (Figure 4b). Using DOPAME, **Cu-BTL** and **Cu-CALB** hybrids maintained the same catechol activity as distilled water, although the decrease in the activity value was found for **Cu-TLL**, in which clearly, the pH conditions are more critical for the Cu-site environment. TYR showed around 5 times higher activity under acidic conditions (Figure 4b) against this substrate compared to DOPA (Figure 4a).

These results could be explained considering the size of the nanoparticles, for example, the synthesis using BTL or CAT as enzymes produced hybrids containing smaller CuNPs than TLL.

Nevertheless, the structural microenvironment created by the particular enzyme, by coordination with copper and amino acid surrounding area, is a key parameter affecting the activity of Cu active sites. Indeed, the isoelectric point (pI) of the enzyme used as a scaffold had also an important influence on the final catalytic capacity of the Cu sites independently of the nanoparticle size in each case. In this way, a higher catechol activity could be detected in DOPA assay when the enzyme used as a scaffold showed a higher pI and enzymes present a pI of 7.2 for BTL, 6.8 for CAT, 6.0 for CALB, and 4.4 for TLL (Figure 4). In this term, at acidic pH, hybrid synthesized using BTL showed the strongest effect on the activity, whereas Cu hybrids created using CALB or TLL even suffered an increase of efficiency compared to the former conditions. When the substrate presented the carboxylic group blocked (DOPAME), only slight changes were observed in almost all hybrids, being the best BTL. Considering the pH effect, in the case of **Cu-CALB** hybrid, an 8 times increase of the catechol activity was observed when the DOPAME reaction was performed at pH 7 (Figure S6).

All of these results demonstrated the key role of the protein environment on the final Cu activity.

### 3.4 Catalase-like Activity

Catalase enzyme is essential for the elimination of the excess of cytoplasmic hydrogen peroxides by converting them to water and molecular oxygen. However, most of the activity described in the literature for other Cu hybrid materials is peroxidase-like activity.^45^

**Figure 5:**
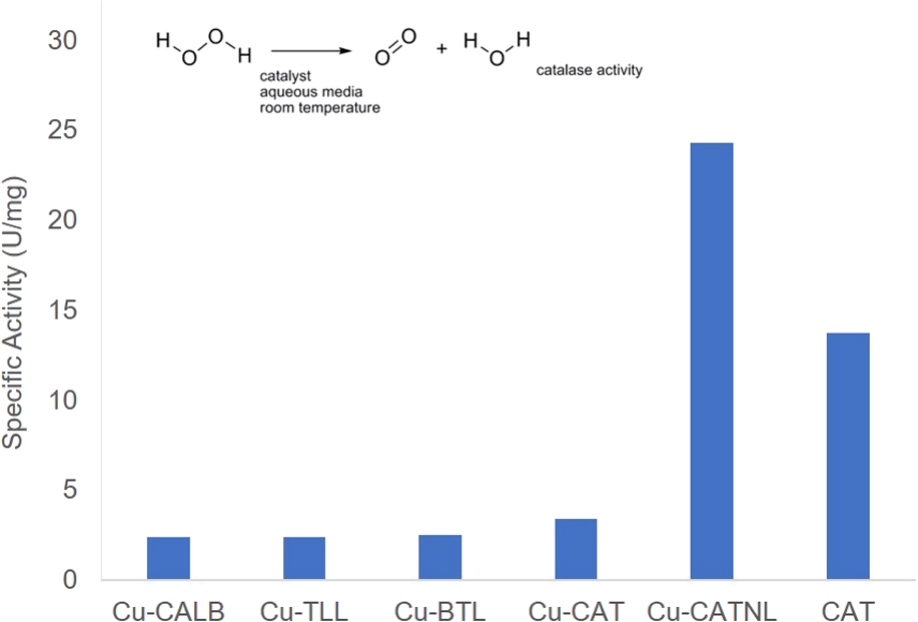
Catalase-like activity of different Enzyme/CuNP hybrids.

Initially, we tested the peroxidase-like activity of these Cu hybrids using the glucose assay and no activity was found for these Cu hybrids (Figure S7). However, the Cu hybrids degraded the hydrogen peroxide to oxygen in distilled water at room temperature (Figure 5). The Cu hybrids prepared using lipases showed similar specific activities, slightly higher for **Cu-BTL** (2.48 U/mg).

However, the degradation of hydrogen peroxide is the natural catalytic reaction of catalase, which under these conditions showed a specific activity of 13 U/mg. Thus, the preparation of a Cu hybrid using this enzyme as a scaffold could allow us to synthesize a nanozyme with a double activity, a synergy between natural and metallic ones.

However, the **Cu-CAT** hybrid showed only a slightly better activity than the previous hybrids, around 3.38 U/mg, which corresponds to around 25% of initial catalase activity of the soluble native enzyme (Figure 5).

One of the explanations could be that it is demonstrated that catalase lost more than 80% activity after lyophilization,^46^ the last step in the preparation of this hybrid.

Therefore, to overcome this drawback, synthesis of the Cu hybrid using catalase as an enzyme scaffold was performed following the previous protocol without the lyophilization step, followed by simple washing and storing the solid resuspended in distilled water.

The characterization of this hybrid without freezing (Figure S8) showed that Cu species were conserved as copper phosphate (XRD analysis), with the formation of homogeneous spherical nanoparticles of diameter 8 nm (TEM analysis), slightly larger than those generated with the freezing step (Figure 2). ICP-OES analysis determined that the amount of copper was also the same.

This **Cu-CAT-NL** hybrid showed much better catalase activity, 8 times higher than that of the lyophilized version (**Cu-CAT**), showing a clear synergy between enzyme and CuNPs in catalyse activity, with double activity compared to free CAT.

The maintenance of the enzyme structure seems to be again important for the CuNPs in addition to the direct intrinsic enzymatic activity.

### 3.5 Dual Activity of Enzyme/CuNP Hybrids

Studies have demonstrated that the presence of oxygen in the media enhanced the catalytic efficiency of tyrosinase or catecholase in the DOPA reaction.

**Figure 6:**
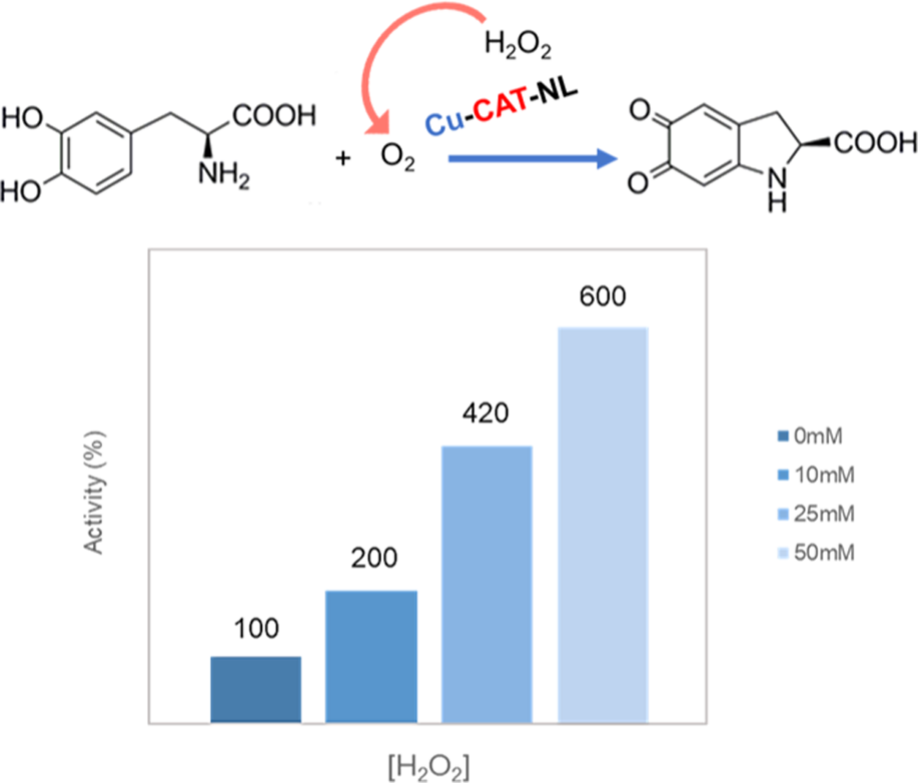
Synergy effect (enzyme plus metal) in the catechol-like activity of **Cu-CAT-NL**. The specific activity of this hybrid in the absence of hydrogen peroxide was considered as 100% activity percentage.

Considering the previous results of **Cu-CAT-NL** in catalase activity in the production of free oxygen in solution from hydrogen peroxide, and their catecholase activity against l-DOPA, slightly lower than the observed for **Cu-CAT**, a tandem system combining l-DOPA and hydrogen peroxide, where oxygen in situ could be reacted with the natural enzyme (catalase) for enhancement of the catechol activity of the CuNP sites, was evaluated (Figure 6).

Thus, the **Cu-CAT-NL** hybrid was used as catalyst in the oxidation of l-DOPA in the presence of different concentrations of hydrogen peroxide (Figure 6).

The results showed how effectively the catecholase activity of the biohybrid increased importantly, up to 6 times, when there was hydrogen peroxide (50 mM) in the medium, due to the synergistic reaction with catalase (enzyme-like scaffold), which increases the presence of oxygen in the medium by degradation of hydrogen peroxide. Lower amounts of hydrogen peroxide also improved the catechol activity of the hybrid Figure 6), but to a lesser extent, showing how the cascade system gave rise to an increase in the initially impaired enzymatic activity due to a combined effect of enzymatic and metallic activities of the biohybrid.

### 3.6 Fenton Catalyst

After these excellent results in modulating tyrosinase and catalase activity, we try to evaluate the effect of the enzyme structure on the Fenton catalysis of the different hybrids.

**Figure 7:**
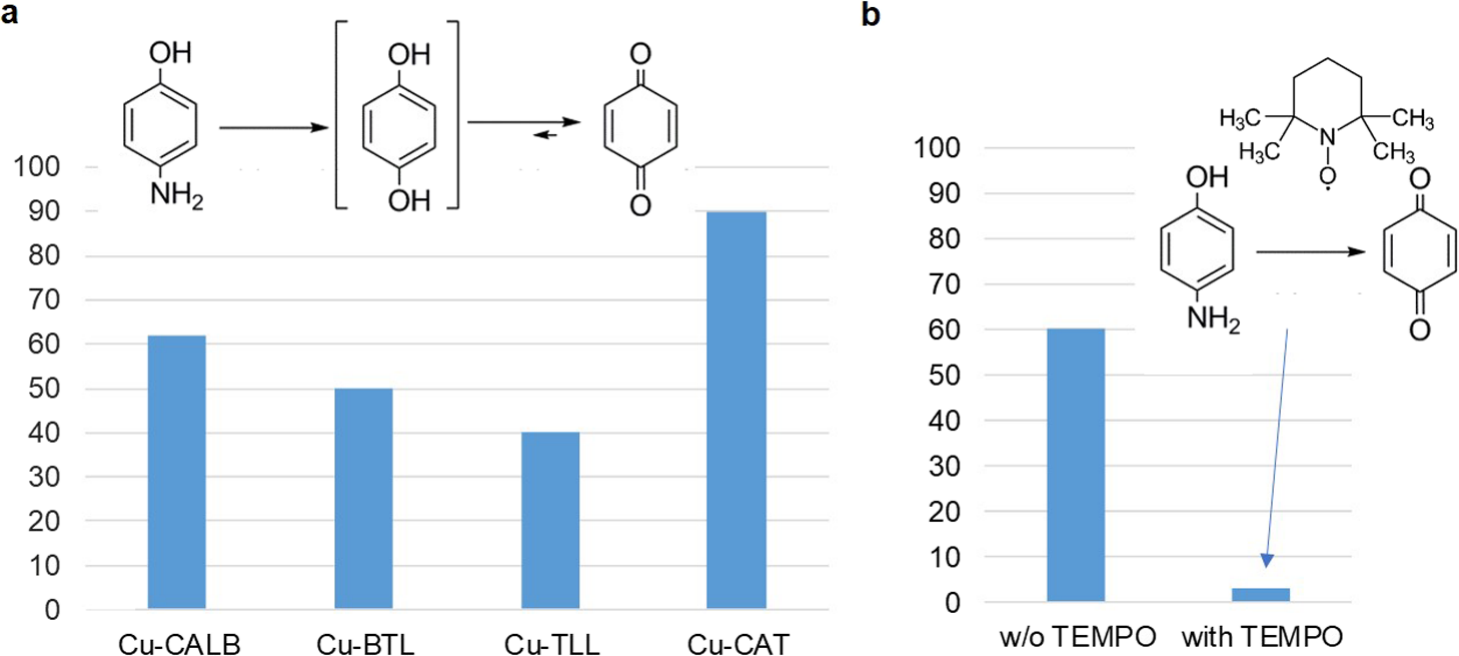
Fenton catalysis in the hydroxylation reaction of *p*-aminophenol to benzoquinone. (a) Conversion of different Cu hybrids after 8 min reaction. Conditions: 100 mg/L of *p*AP in 10 mL of distilled water, 1%, v/v H_2_O_2_, and 3 mg of Cu hybrid at room temperature. (b) Reaction catalyzed by **Cu-CALB** with or without TEMPO.

The selective hydroxylation of *p*-aminophenol to benzoquinone using hydrogen peroxide as a green oxidant was used (Figure 7). The Fenton process was observed with these Cu hybrids. A clear tendency was observed in the case of using lipases as an enzyme scaffold, where higher conversion is achieved for smaller Cu nanoparticles. However, the best result was found using CAT as an enzyme, with almost 90% conversion in 7 min, which indicates the influence of the enzyme structure together with the nanoparticle size.

Furthermore, to demonstrate the Fenton process mechanism of the reaction, with radical OH^•^ formation, the reaction was also performed in the presence of TEMPO using **Cu-CALB** as a catalyst (Figure 7b). Under these conditions, only 3% conversion was observed after 7 min (60% without adding TEMPO) with a clear decrease in the reaction process in the reaction profile.

### 3.7 Assessing Cell Metabolic Activity of the Enzyme/CuNP Hybrids on Cancer Cells

One of the emerging applications of nanozymes focuses on their in vitro nanocatalytic therapeutic efficiency.

**Figure 8:**
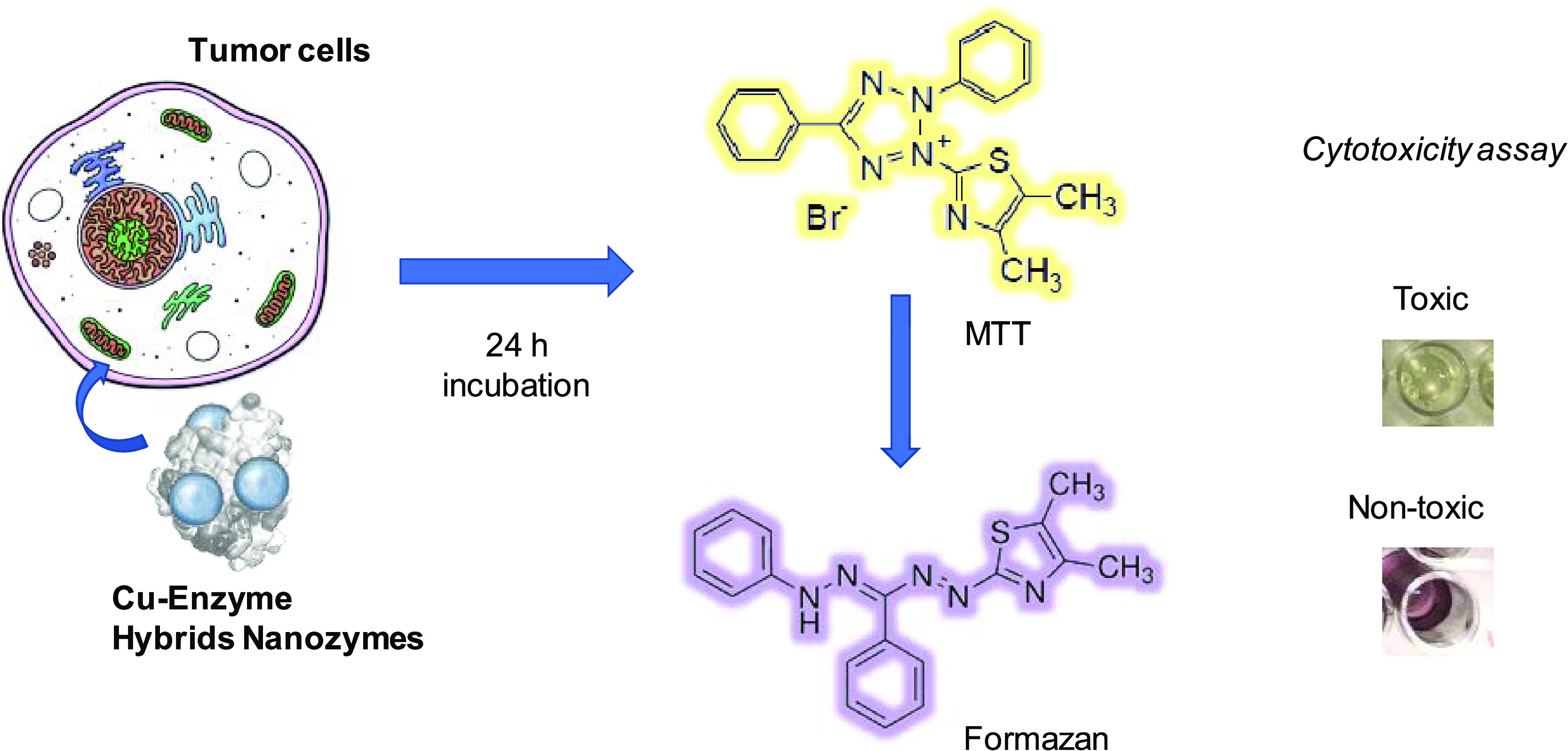
Schematic of the MTT assay.

In this term, in vitro cytotoxic activity of the Cu hybrids has been evaluated in two different cancer cell lines, HeLa (human cervical cancer) and HT29 (human colon cancer cells). Different concentrations of the Cu hybrids were used in the assay after 24 h incubation. The cell metabolic activity was determined using the colorimetric 3-(4,5-dimethylthiazol-2-yl)-2,5- diphenyl tetrazolium bromide or methyl thiazole tetrazolium bromide (MTT) assay (Figure 8).

The essence of this assay is based on the metabolic reduction of (MTT), which is carried out by the enzyme mitochondrial succinate dehydrogenase from metabolically active mitochondria cells. This enzyme transforms MTT from a yellow hydrophilic soluble compound to a blue hydrophobic insoluble compound (formazan) (Figure 8) by cleavage of the tetrazolium ring by dehydrogenase enzymes. Consequently, this transformation enables the mitochondrial function of the treated cells to be determined. The product of the reaction, formazan, is retained in the cells and can be released by the solution thereof. The ability of the cells to reduce MTT is an indicator of the integrity of mitochondria.

**Figure 9:**
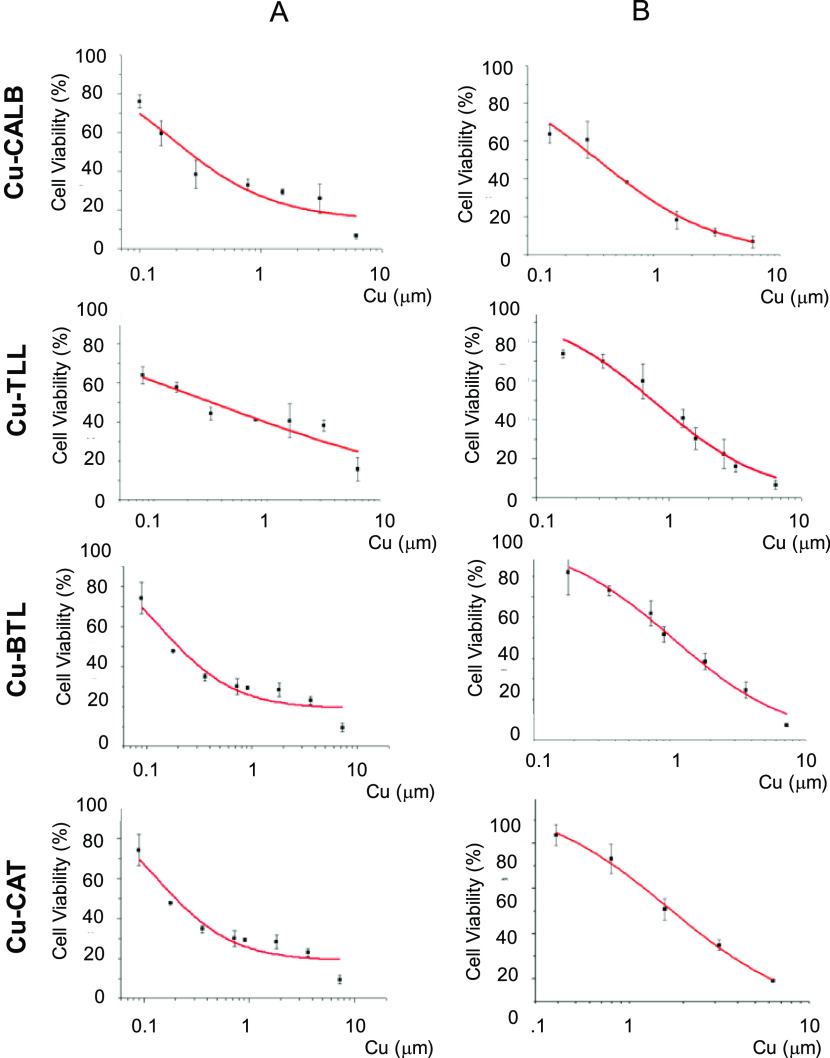
Curve cell viability in cancer cells calculated in μM concentration of Cu of Cu hybrids: (A) HeLa cells and (B) HT29 cells.

The viability according to Cu content in hybrids is represented in Figure 9. Differences in cell viability depending on the Cu hybrids used, as well as different effect depending on the cancer cell lines, can be found, showing higher cytotoxicity against HeLa cells (Figure 9). **Cu-CALB** and **Cu-TLL** showed the best cytotoxicity at a lower concentration (0.1 lower concentrations).

The concentration of copper in which 50% of initial activity is reached (LC50) (Table 1) was determined from the fitting of these curves in Figure 9.

**Table 1:** LC50 (μM) Values of the Different Cu–Enzyme Hybrids on HeLa and HT29 Cell Lines

Cu–enzyme hybrid	HeLa LC50 Cu (μM)	HT29 LC50 Cu (μM)
**Cu-CALB**	0.18	0.37
**Cu-BTL**	0.13	1.05
**Cu-TLL**	0.33	0.8
**Cu-CAT**	1.25	1.65

HeLa cells seemed to be more sensitive to Cu than HT29 cells, up to 10 times in the case of **Cu-BTL** hybrid (0.13 and 1.05 μM of Cu, respectively), which showed the lowest LC50 values in HeLa cells (Table 1). In the case of **Cu-CAL-B**, this showed the lowest LC50 values (0.37 μM of Cu) in HT29 cell lines also being one of the best in cytotoxicity against HeLa cells. **Cu-CAT** exhibited the highest LC50 values in HeLa and HT29 (1.25 and 1.65 μM of Cu, respectively) (Table 1). **Cu-TLL** (0.33 and 0.8 μM of Cu) is in between the highest and lowest values. The different enzymes used for preparing the hybrid were tested, and no cytotoxicity was found (Figure S9).

Thus, **Cu-CALB** hybrid, which presents the smallest size in Cu nanoparticles, seemed to be the enzyme-like derivative with the highest cytotoxic activity in the tested cell cultures. It has been reported that Cu can generate reactive oxygen species (ROS) in the cell and ROS increment causes DNA damage.^47,48^ According to the results of this work, our Cu–enzyme hybrids exhibited antitumor properties, as other Cu compounds previously described in the literature probably based on the oxidative enzyme-mimicking capacity (such as xanthine oxidase- or monoamine oxidases-like activity) of the Cu nanoparticles on them.

### 3.8 Protein Structural Effect of Enzymes as a Scaffold in the Formation of Enzyme/CuNP Hybrids

The use of an enzyme in the nanozyme synthesis has a key role in the stabilization and formation of homogenously distributed copper nanoparticles and their stabilization on the protein network.

Indeed, the formation of hybrid copper nanoparticles first undergoes by a rapid coordination between particular amino acid residues of the enzyme with the copper ions, generating enzyme–Cu(ii) intermediates (Figure 1). It is well described that copper ions have very good coordination capacity, especially with carboxylic groups (Asp, Glu) and also with amino groups, mainly those containing histidine residues. Interaction with cysteine residues may also be possible.

However, the three-dimensional environmental area around these coordinated groups, which can be different depending on the enzyme, seems to play a critical role. In this case, we have seen that employing different enzymes, the same Cu species were formed.

**Figure 10:**
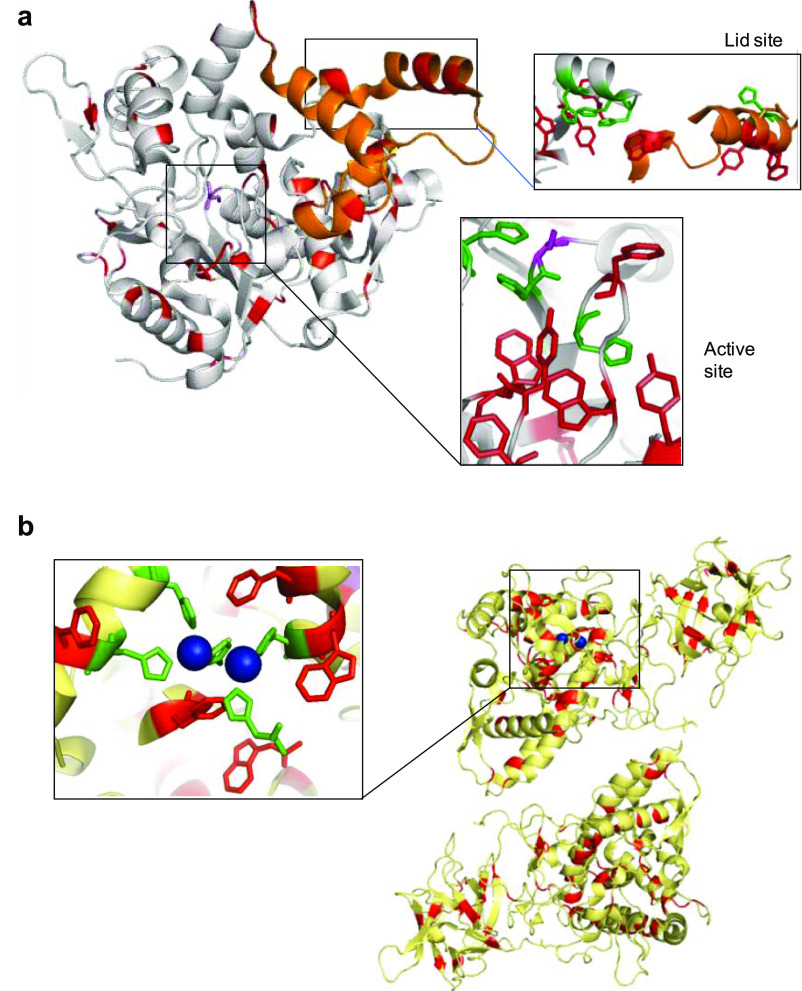
Crystal structure of *B. thermocatenulatus* lipase and mushroom tyrosinase. (a) Cartoon of the crystallized *B. thermocatenulatus* lipase (BTL) with aromatic amino acids (Trp, Tyr, Phe) in red, histidine residues in green, the catalytic serine in magenta, and the oligopeptide lids in orange. (b) Cartoon of crystallized mushroom tyrosinase (TYR) with aromatic amino acids (Trp, Tyr, Phe) in red, histidine residues in green, and Cu atoms in blue. The protein structures were obtained from the Protein Data Bank (PDB code: 2W22 (BTL) and 2Y9W TYR), and the picture was created using Pymol v.0.99.

Therefore, bioinformatic analyses were performed to evaluate the structural environmental effect of the enzymes on the enzyme-like activity of the CuNP sites in different hybrids (Figures 10, S10, and S11).

In particular, in the case of catechol-like activity, Cu hybrid synthesized using BTL showed the highest enzymatic activity in all cases, being also affected by the pH, especially using l-DOPA (Figure 4).

This is a specific lipase, which presents an extremely hydrophobic area surrounding the active site, where also is located an area containing His residues (imidazole groups), where Cu can be coordinated (Figure 10a). Metal-binding sites in proteins are commonly formed from loops because these regions are reasonably tolerant to sequence modifications outside of coordinating residues.

Furthermore, this lipase presents another interesting area, near one of the two lids involved in the catalytic mechanism of the enzyme,^49^ where we can find a perfect trihistidine pocket (Figure 10a) similar to that present in the natural tyrosinase (Figure 10b).^44^ Both His pockets are surrounded by different aromatic amino acid residues (e.g., Trp, Phe, or Tyr), which are important in the substrate stabilizing the catechol group near the Cu binding position on the protein for permitting the catalytic transformation, as occurred in the natural enzymes. It has been demonstrated that lipase mechanism has a strong influence on pH, especially the thermoalkalophilic one,^49^ so results showing loss of catechol-like activity in acidic pH or a different effect using DOPAME as a substrate clearly demonstrate that the typical structural form of this lipase has an influence on the final CuNP site catalysis.

The Enzyme/CuNP hybrids synthesized using catalase involved the use of a multimeric enzyme, with four identical subunits of 80 kDa. Each subunit presents 20 histidine residues, for the successful coordination of Cu ions and a large number of aromatic amino acid residues (Figure S10). This could indicate, although unfortunately the structure of catalase from *A. niger* is still not solved, that structural environment (similar to the BTL case) could be involved in the final good activity of CuNP sites.

An opposite result was obtained with TLL, and even no catechol activity was found under some condition. This lipase has a huge trend to form aggregates—even of eight molecules—that make it, in aqueous solution, a complex enzyme.^41^ This could be the reason why TLL produced larger nanoparticles compared with CALB and lipase with the same molecular weight in monomeric form^50^ (Figure S11). This property has an influence on the natural activity of the enzyme and clearly is also a limitation for the catechol-like activity.

### 3.9 Stability of the Enzyme/CuNP Nanozymes

**Figure 11:**
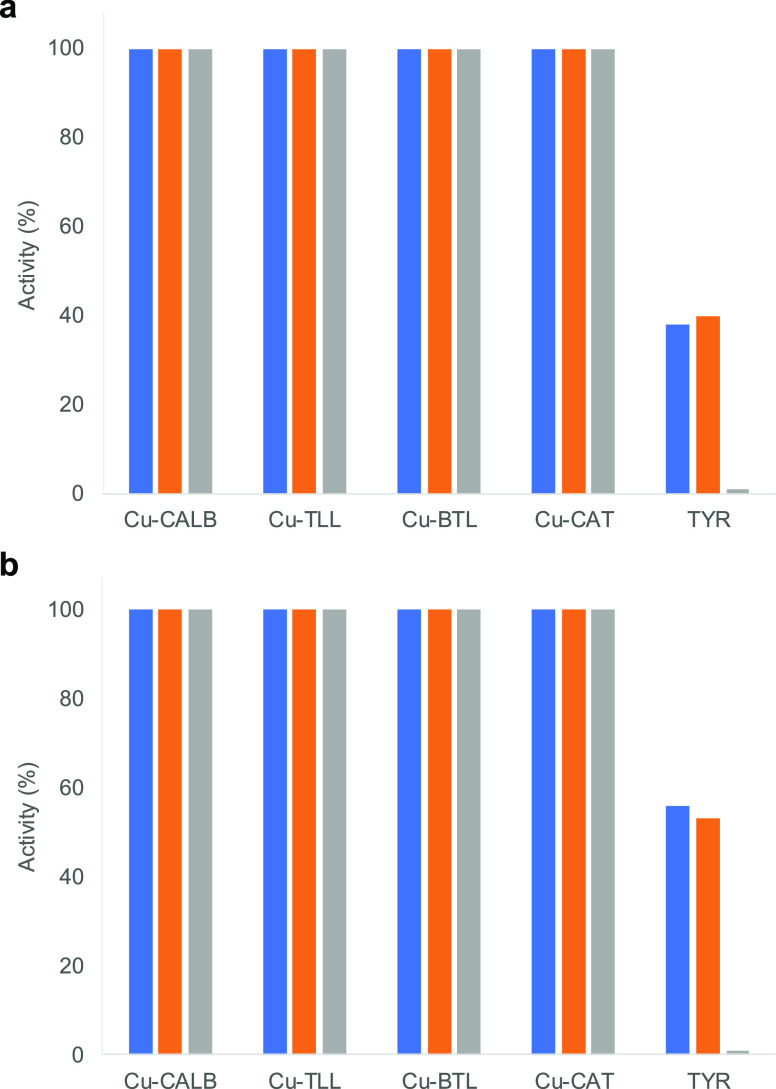
Stability of different Enzyme/CuNP hybrids vs tyrosinase (TYR) in catechol-like activity. (a) Oxidation reaction of l-DOPA. (b) Oxidation reaction of l-DOPAME. Experiments were performed at 37 °C (blue column), 40% (v/v) acetonitrile (orange column), and 37 °C and 40% (v/v) acetonitrile (gray column).

Another important property of enzymes is stability. In particular, tyrosinase has been detected as not so stable under biological conditions. In this point, the stability of the most active Cu hybrids, in comparison to TYR, was evaluated at different temperatures and in the presence of a co-solvent (Figure 11). TYR was rapidly inactivated at 37 °C, and conserving only 38% of the initial activity after 1 h incubation, using DOPA as a substrate (Figure 11a). However, the stability of TYR was better using DOPAME as a substrate, conserving more than 50% activity after 1 h incubation at 37 °C (Figure 11b). The stability of this enzyme in the presence of 40% (v/v) acetonitrile at room temperature was also low, conserving around 45% initial activity value after 1 h incubation.

The combination of both elements, temperature and co-solvent, caused a complete inactivation of the enzyme after 1 h incubation (Figure 11).

The different Cu hybrids synthesized conserved 100% of their catechol-like activity after 1 h incubation under any of these conditions (Figure 11), demonstrating that these nanozymes could represent an alternative to sensitive enzymes in many processes. These enzymes used as a scaffold were stable under these experimental conditions (data not shown), which could be important in terms of maintaining the three-dimensional structure, which was also observed in the circular dichroism and fluorescence experiments (Figure 3).

**Figure 12:**
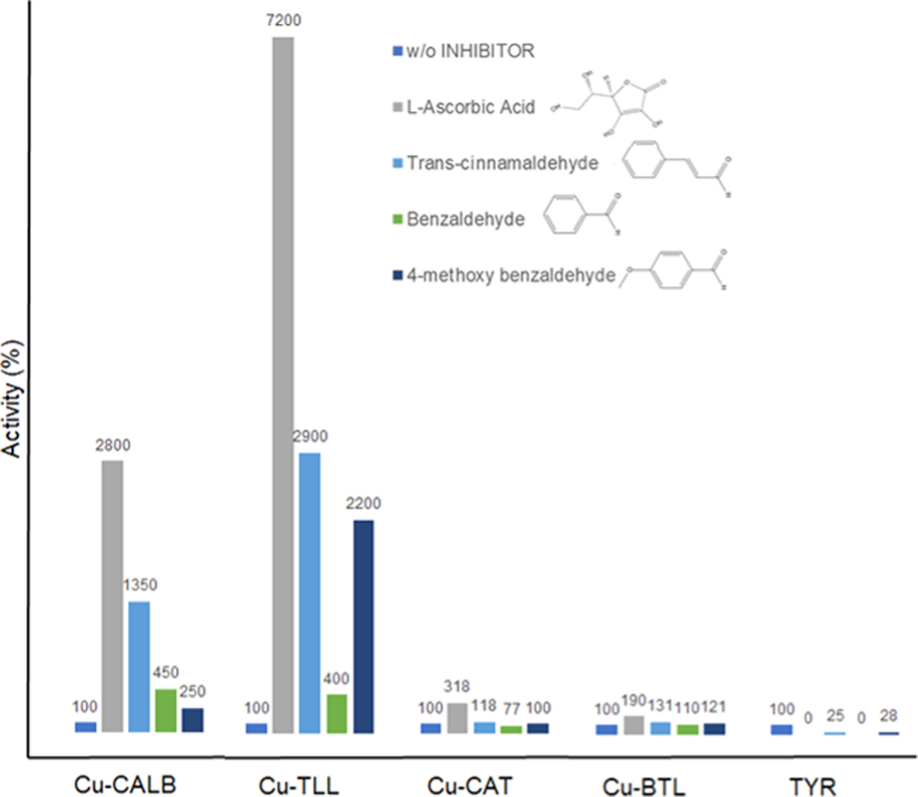
Tyrosinase activity of different Enzyme/CuNP hybrids vs TYR in the presence of different tyrosinase inhibitors. l-DOPA in distilled water assay was used. Condition: 1 mM l-DOPA in 2 mL of distilled water at rt. The inhibitors were added at 1 mM.

In terms of stability, one of the disadvantages of enzymes is the possible inhibition by substrates. In particular, mushroom tyrosinase (TYR) activity has been demonstrated to be inhibited by different conjugated or aromatic compounds. Here, four of these known substrates^51^ (l-ascorbic acid, *trans*-cinnamaldehyde, benzaldehyde, or 4-methoxy benzaldehyde) have been added in the catechol assay and the activity of the Cu hybrids has been measured (Figure 12).

TYR activity was rapidly inhibited in the presence of these substrates, only conserving some activity (25% of initial activity) against *trans*-cinnamaldehyde and 4-methoxybenzaldehyde (Figure 12).

However, enzyme/CuNP hybrids did not suffer any inhibition; indeed, in some cases, hyperactivation was observed in the presence of these molecules (Figure 12). Very surprising could be the increase of catechol-like activity of hybrids synthesized using CALB and especially using TLL. In particular, Cu hybrids containing TLL improved its previous catechol activity 72-fold in the presence of ascorbic acid, while the hybrid with CALB showed a 28-fold improvement (Figure 12). However, this effect could be explained considering the reported increase in hydrolytic activity of CALB in the presence of aromatic or conjugated compounds^52^ or in the case of TLL, whose activity was increased more than 600 times in the presence of a small amount of CTAB (cationic detergent).^53^ Therefore, these results seem to show that the protein structure has an important influence changing the catalytic capacity of the Cu active sites on it.

## 4 Conclusions

Enzyme/CuNP hybrids with controlled nanoparticle sizes and environment have been successfully synthesized using different enzymes. The difference between enzymes, where we can use three different lipases with a particular catalytic mechanism, and the use of a supramolecular tetrameric catalase demonstrates the important effect of the structure on the final catalytic properties as nanozymes.

In particular, the oxidase-like catalytic activity of these copper nanozymes was rationally modulated by the enzyme used as a scaffold with important ability to mimic a unique enzyme activity.

For example, the tyrosinase-like activity of these Cu hybrids was clearly modulate by the enzymes, and **Cu-BTL** was the one showing very high activity against l-DOPA or l-DOPAME oxidation, demonstrating the role of the enzyme used. Importantly, this nanozyme showed extremely high stability under conditions where natural tyrosinase was completely inactive.

In the catalase-like activity, a synergic activity between the Novozymes catalase and CuNPs created on using this enzyme (**Cu-CAT-NL**) allows us to achieve a nanozyme with enhanced activity with respect to the natural biocatalyst, with the preservation of the three-dimensional structure of the enzyme as a scaffold being quite critical, which is not observed when lyophilization step was used in the Cu hybrid creation (**Cu-CAT**).

Furthermore, a very interesting dual activity was found to increase the catechol-like activity of the CuNPs in **Cu-CAT-NL**. In this case, the presence of hydrogen peroxide in the l-DOPA assay allowed us to greatly improve this activity for the CuNPs by the enzymatic activity in the hybrid (CAT), which generates in situ oxygen in the process. This could be interesting for the design of an l-DOPA biosensor for the detection of tyrosinase, which has been found at elevated amounts on melanoma cancer cells.

For the generation of radical hydroxyl species, Fenton catalyst application of the hybrids demonstrated a clear tendency in lipases as the scaffold used, where the best result was found when smaller nanoparticles were obtained. In this reaction, **Cu-CAT** was however the most reactive one.

This typical nanozyme activity was evaluated in the cytotoxicity of these hybrids in different human cancer cell lines, and **Cu-CALB**—which presents the smallest enzyme with the smallest nanoparticle sizes—showed the best antitumor activity.

Therefore, these results showed that different nanozymes of the same Cu species with a tailor-made enzyme-like activity could have potential therapeutic and diagnostic applications.

## Supporting Information Available

TEM figures, XRD pattern, SEM figures, enzymatic-like activity results, MTT assay, protein sequences data, and ICP analysis results (PDF)
