# Supplementary material for: Enzyme/Nanocopper Hybrid Nanozymes: Modulating Enzyme-like Activity by the Protein Structure for Biosensing and Tumor Catalytic Therapy
Source: ACS Appl Mater Interfaces. 2021 Jan 21;13(4):5111–24. doi: 10.1021/acsami.0c20501 (PMC8486171; doi:10.1021/acsami.0c20501)
Supplement: Supplementary file 1 — am0c20501_si_001.pdf [file am0c20501_si_001.pdf]

Supporting Information

Enzyme/NanoCopper hybrid Nanozymes: Modulating Enzyme-like Activity by the protein structure for biosensing and tumor catalytic therapy

Noelia Losada-Garcia,[1] Ana Jimenez-Alesanco,[3] Adrian Velazquez-Campoy,[2-6] Olga Abian[3-7] and Jose M. Palomo[1]*

[1]Department of Biocatalysis, Institute of Catalysis (CSIC)

Address: c/marie curie 2, cantoblanco campus UAM, 28049, Madrid, Spain

e-mail: josempalomo@icp.csic.es

[2] Fundación ARAID, Gobierno de Aragón, Zaragoza, Spain;

[3]Instituto de Biocomputación y Física de Sistemas Complejos, Joint Units IQFR-CSIC-BIFI, and GBsC-CSIC-BIFI, Universidad de Zaragoza, Spain

[4]Fundación Instituto de Investigación Sanitaria de Aragón (IIS Aragon), Zaragoza, Spain

[5]Centro de Investigación Biomédica en Red en el Área Temática de Enfermedades Hepáticas y Digestivas (CIBERehd), Madrid, Spain

[6]Departamento de Bioquímica y Biología Molecular y Celular, Universidad de Zaragoza, Zaragoza, Spain

[7]Instituto Aragonés de Ciencias de la Salud (IACS), Zaragoza, Spain

Peroxidase-like activity assay

Glucose (0.5 mL, 1M), 2,2′-Azino-bis(3-ethylbenzothiazoline-6-sulfonic acid) di ammonium salt (ABTS) (0.1 mL of 1mg/mL solution in water) and horseradish peroxidase (HRP) (0.1 mL of 2 mg/mL solution in buffer phosphate 0.1M) or 100 µL of nanohybrids —from a solution of 1 mg hybrid/ml distilled water— were added to a 2 mL of 0.1M buffer sodium phosphate pH 6 at room temperature. The absorbance of solution was measured at 414 nm in a JASCO V-730 UV-spectrophotometer.

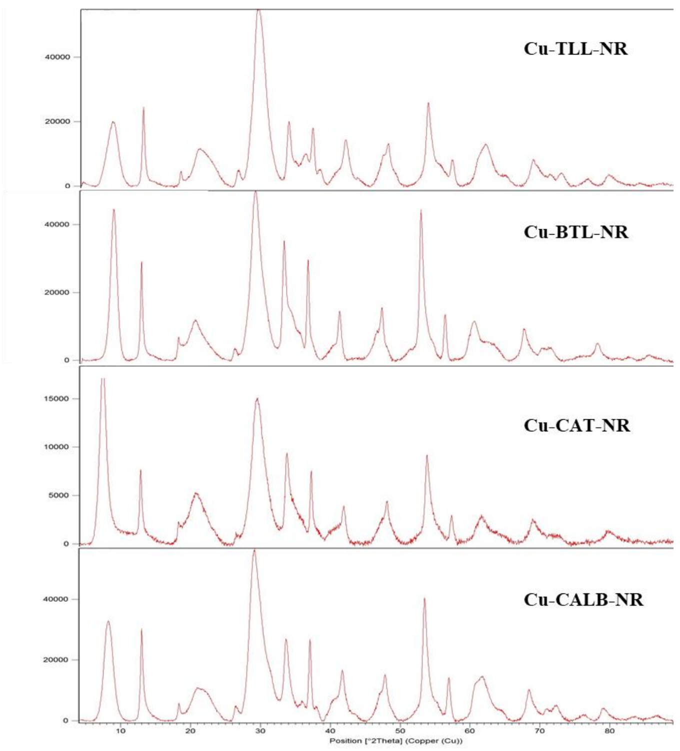

Figure S1. XRD pattern of Enzyme/CuNPs hybrid. Wide-angle XRD further displayed characteristic peaks of $Cu_3(PO_4)_2$ (matched well with JCPDS card no. 00-022-0548).

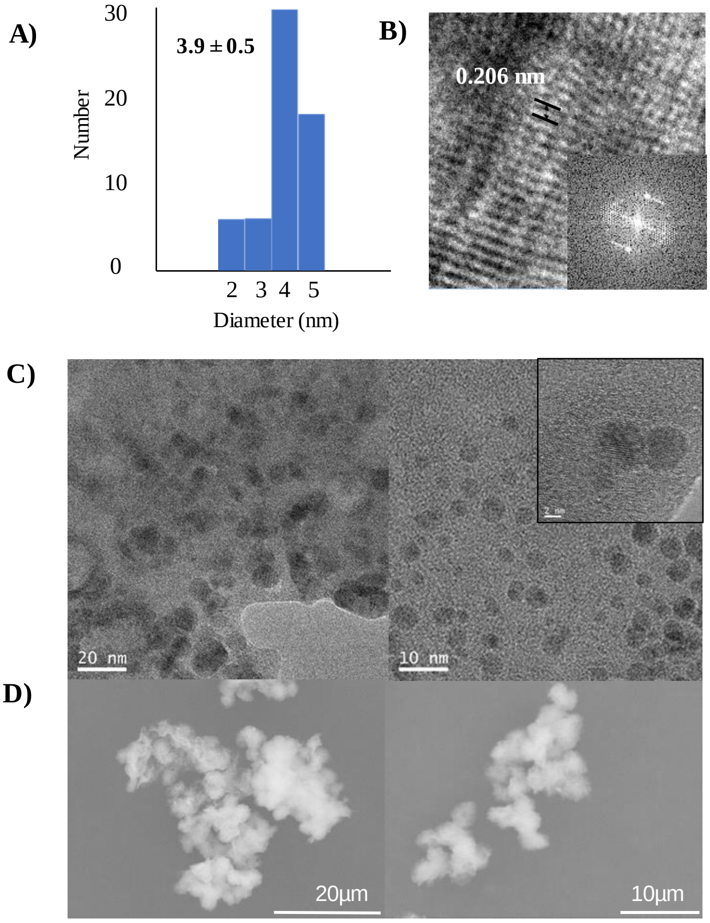

Figure S2. Cu-CALB hybrid. A) Particle size distribution profile; B) Crystalline section with representative lattice fringe (inset FFT), C) TEM and HRTEM images; D) SEM.

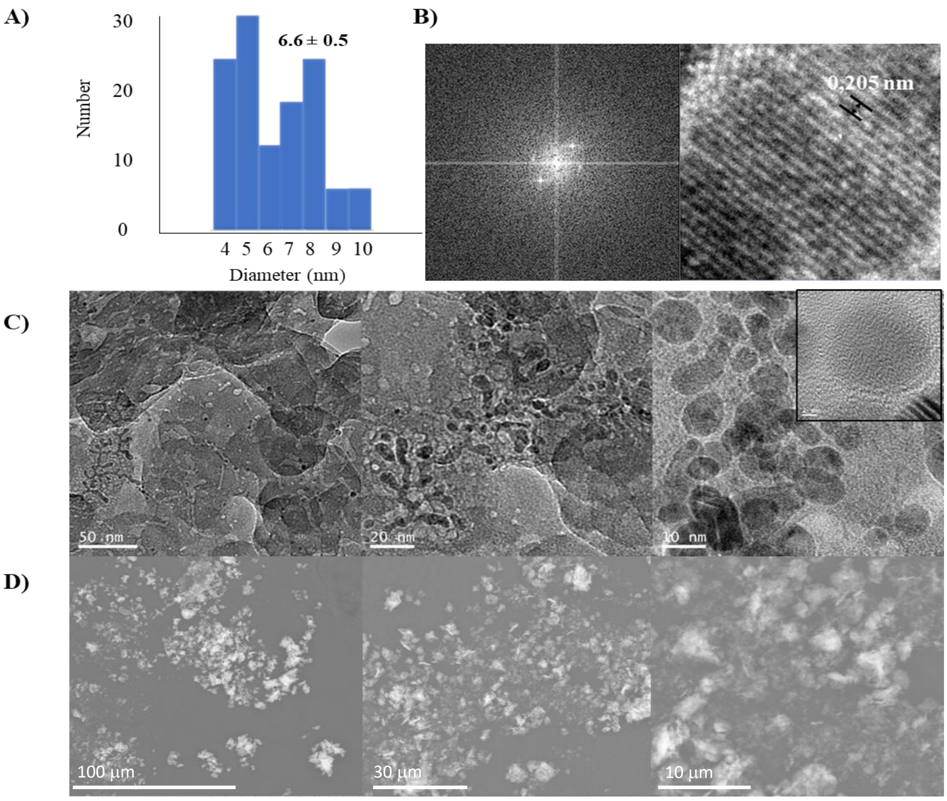

Figure S3. Cu-BTL hybrid. A) Particle size distribution profile; B) Crystalline section with representative lattice fringe and its corresponding FFT, C) TEM and HR-TEM images; D) SEM images.

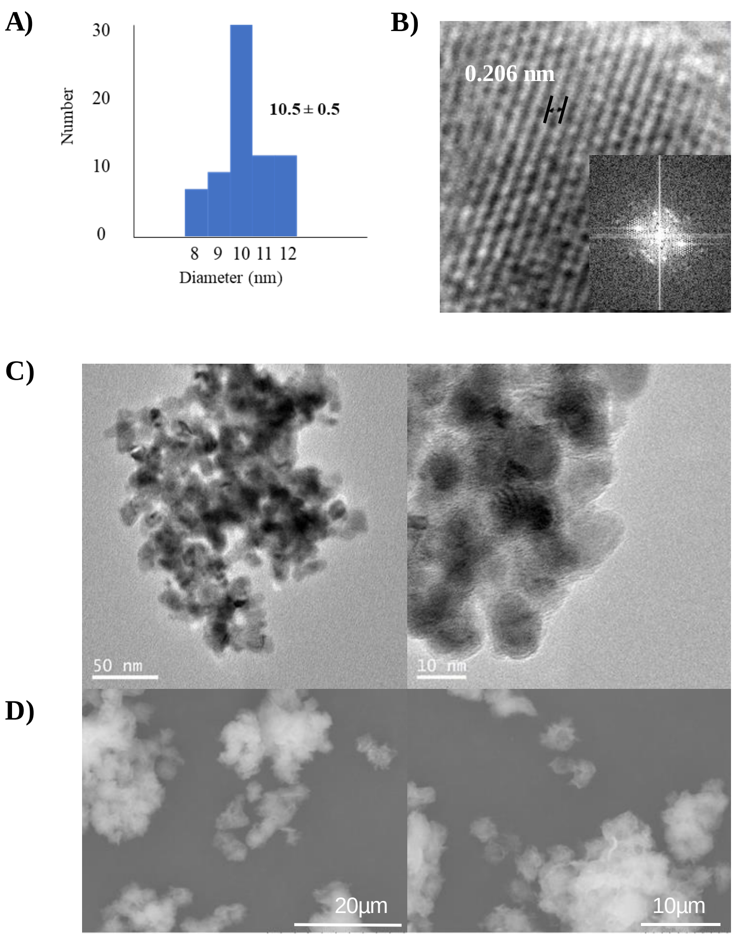

Figure S4. Cu-TLL hybrid. A) Particle size distribution profile; B) Crystalline section with representative lattice fringe (inset FFT), C) TEM and HRTEM images; D) SEM

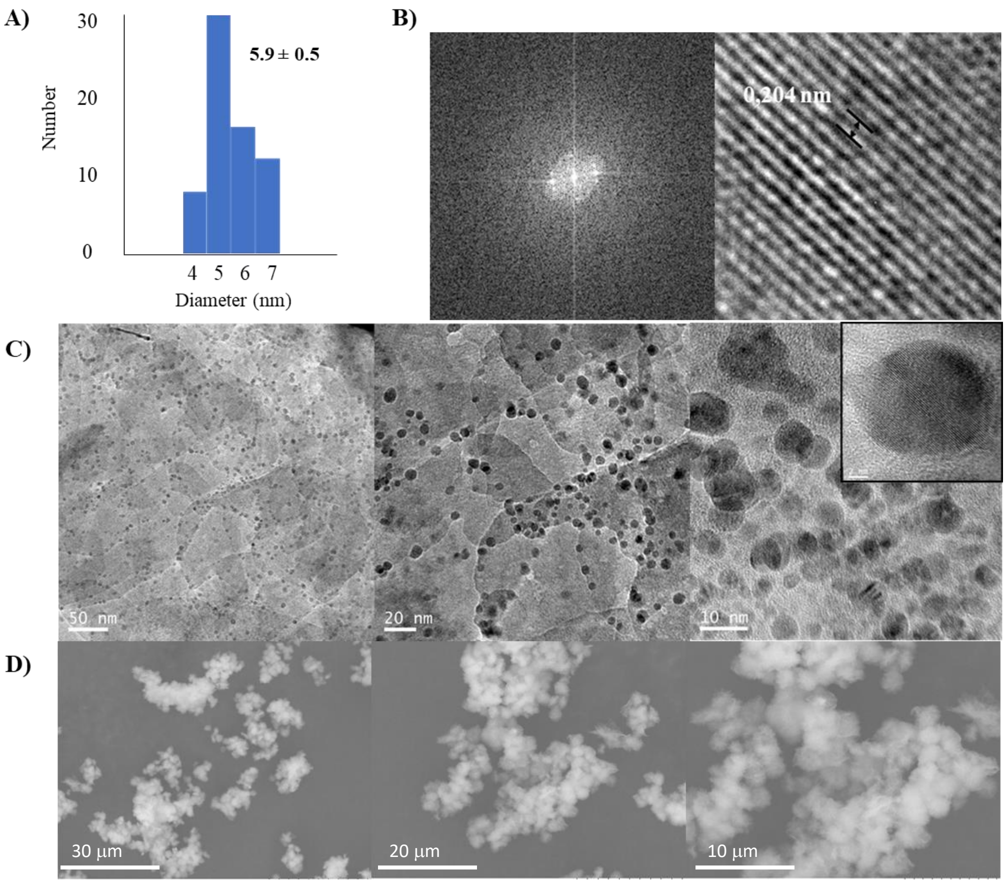

Figure S5. Cu-CAT hybrid. A) Particle size distribution profile; B) Crystalline section with representative lattice fringe and its corresponding FFT; C) TEM and HR-TEM images; D) SEM.

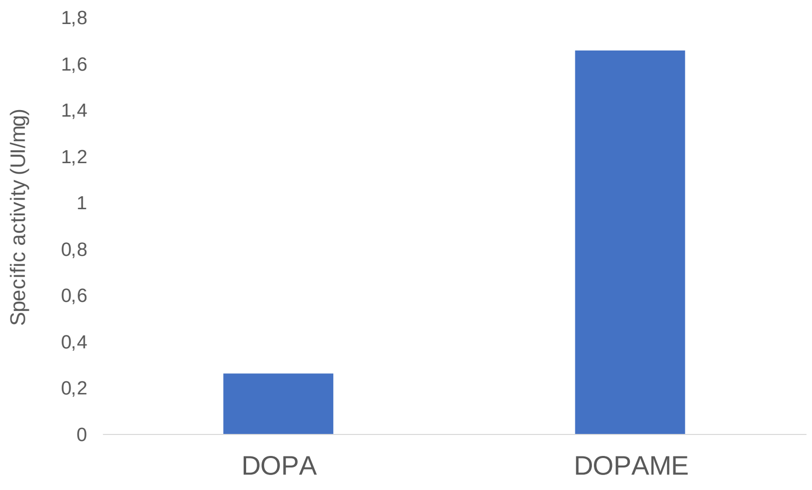

Figure S6. Tyrosinase-like activity of Cu-CAL-B hybrid. Oxidation at pH 7 was expressed in values of specific activity (U/mg).

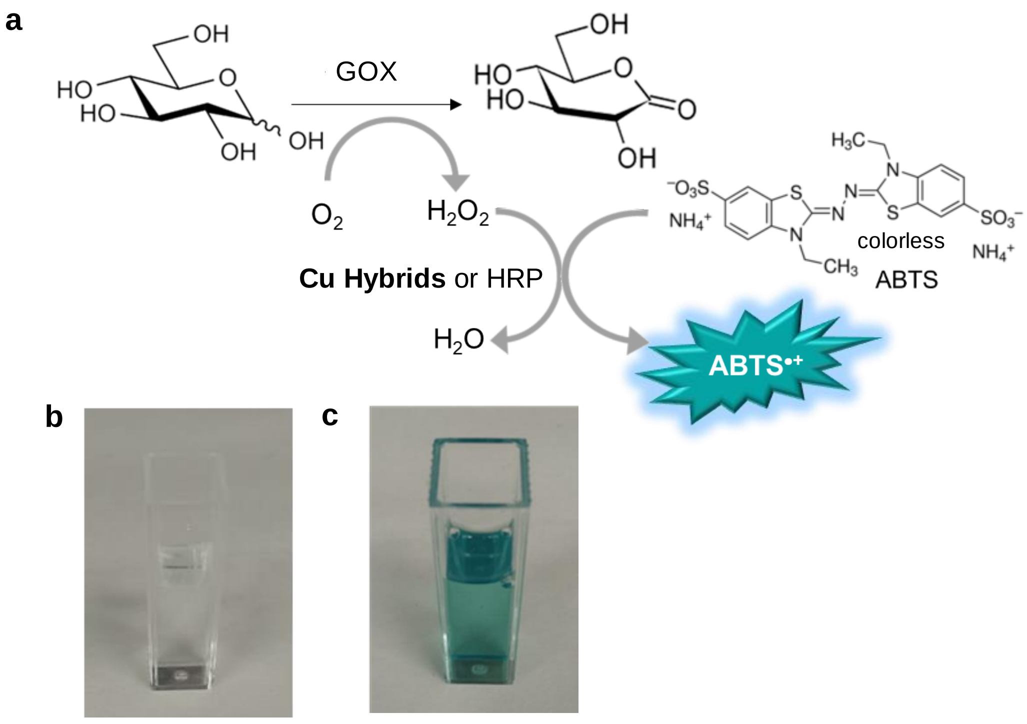

Figure S7. Evaluation of peroxidase-like of Enzyme/CuNPs hybrids. (a) General reaction cascade biosensing scheme. (b) Assay using Cu hybrids as peroxidase mimics. (c) Assay using horse reddish peroxidase (HRP).

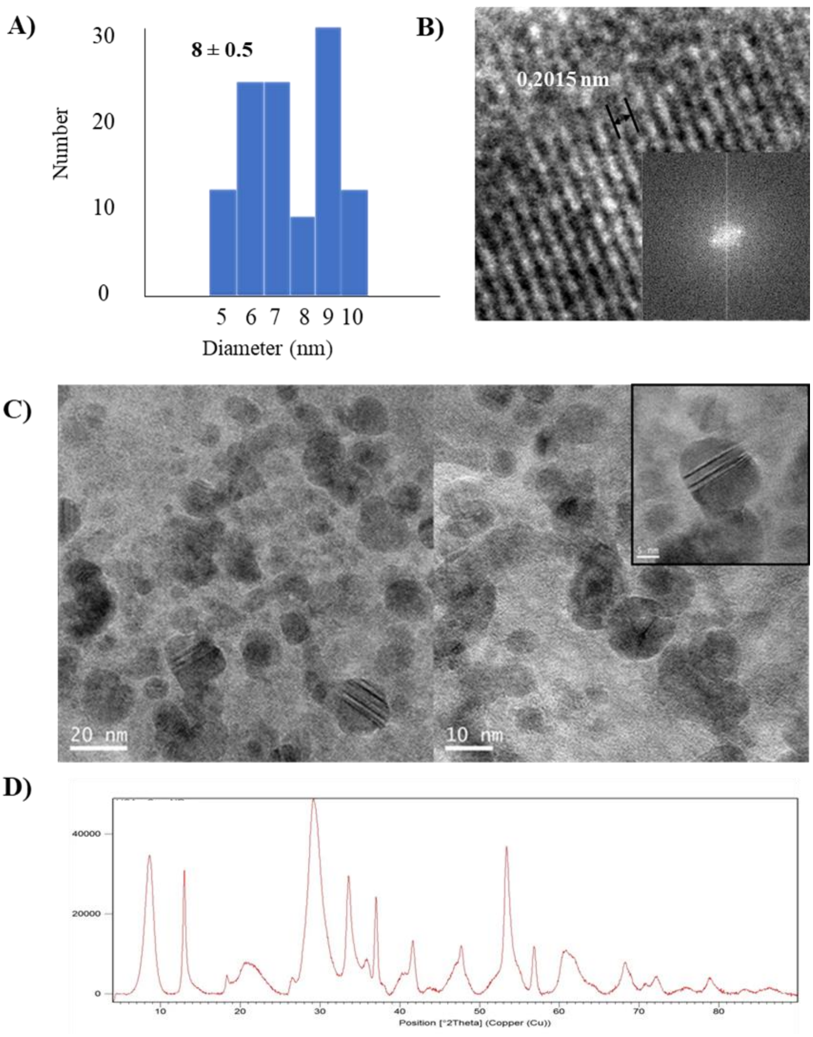

Figure S8. Cu-CAT-NL hybrid. A) Particle size distribution profile; B) Crystalline section with representative lattice fringe and its corresponding FFT.; C) TEM and HR-TEM images. D) Spectrum of XRD. Wide-angle XRD further displayed characteristic peaks of $Cu_3(PO_4)_2$ (matched well with JCPDS card no. 00-022-0548).

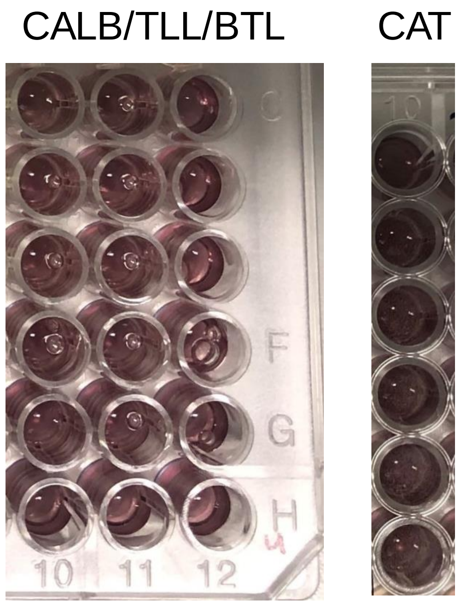

Figure S9. MTT assay of free enzymes.

```
          10         20         30         40         50
MRHFWLLPAV AGIAGAQCPY LSGEMSFTQE QDNAGDTIEV TEQPIDNTLY
          60         70         80         90        100
VNDTGSYMTT DFGTPISDQT SLKAGPRGPT LLEDFIFRQK LQRFDHERVP
         110        120        130        140        150
ERVVHARGAG AYGTFKSYAD WSNVTAADFL SANDKETPMF CRFSTVVGFR
         160        170        180        190        200
GSVDTARDVH GHACRFYTDE GNYDIVGINF APFFIQDAIQ FPDLVHAIKP
         210        220        230        240        250
MPNNEIPQAA TAHTSAWDFF SQQSTALHSA LWLMSGNGIP RSFRHMNGYG
         260        270        280        290        300
VHSFRFVAANGTSKVVRYRWKSQQGVASLV WDEAQAAAGK NSDYHRQDLY
         310        320        330        340        350
NAIANGHYPK YELQAQIMDE ADMLRFGFDL LDPTKLVPEE VVPYTPLGMM
         360        370        380        390        400
ELNANPTNYF AEVEQAGFQP GHVVPGIDFT DDPLLQGRLF SYLDTQLTRH
         410        420        430        440        450
GGPNFEQIPV NRPRKPVHNN NRDGFGQQQI PTNNWAYTPN SMSNGYPMQA
         460        470        480        490        500
NQTQGHGFFT APYRYASGHL VRQTSPTFND HWSQPAMFWN SLIPAEQQMV
         510        520        530        540        550
VNAIVFENSK VNSPHVRKNV VNQLNMVNNN LAVRVARGLG LDEPSPNPTY
         560        570        580        590        600
YTSNKTSNVG TFGKPLLSIE GLQVGFLASN SHPESIKQGQ AMAAQFSAAG
         610        620        630        640        650
VDLNIVTEAY ADGVNTTYAL SDAIDFDALI IADGVQSLFA SPALANQMNS
         660        670        680        690        700
TATSTLYPPA RPFQILVDSF RYGKPVAAVG SGSVALKNAG IDSSRSGVYT
         710        720        730
GSSETTEKIA KEVLEGLYTF RFVDRFALDE
```

Figure S10. Amino acids sequence of CAT (Catalase from *Aspergillus niger*). Asp and Glu marked in yellow. His in green. Trp marked in blue. Sequence was obtained as reported.[1] Iron-protein binding at 392 amino acid position (brown).

A) 10 20 30 40 50

MALPSGSDPA FSQPKSVLDA GLTCQGASPS SVSKPILLVP GTGTTGPQSF

60 70 80 90 100

DSNWIPLSTQ LGYTPCWISP PPFMLNDTQV NTEYMVNAIT ALYAGSGNNK

110 120 130 140 150

LPVLTWSQGG LVAQWGLTFF PSIRSKVDRL MAFAPDYKGT VLAGPLDALA

160 170 180 190 200

VSAPSVWQQT TGSALTTALR NAGGLTQIVP TTNLYSATDE IVQPQVSNSP

210 220 230 240 250

LDSSYLFNGK NVQAQAVCGP LFVIDHAGSL TSQFSYVVGR SALRSTTGQA

260 270 280 290 300

RSADYGITDC NPLPANDLTP EQKVAAAALL APAAAAIVAG PKQNCEPDLM

310

PYARPFAVGK RTXSGIVTPS L

B)

10 20 30 40 50

MRSSLVLFFV SAWTALASPI RREVSQDLFN QFNLFAQYSA AAYCGKNNDA

60 70 80 90 100

PAGTNITCTG NACPEVEKAD ATFLYSFEDS GVGDVTGFLA LDNTNKLIVL

110 120 130 140 150

SFRGSRSIEN WIGNLNFDLK EINDICSGCR GHDGFTSSWR SVADTLRQKV

160 170 180 190 200

EDAVREHPDY RVVFTGHSLG GALATVAGAD LRGNGYDIDV FSYGAPRVGN

210 220 230 240 250

RAFAEFLTVQ TGGTLYRITH TNDIVPRLPP REFGYSHSSP EYWIKSGTLV

PVTRNDIVKI EGIDATGGNN QPNIPDIPAH LWYFGLIGTC L

Figure S11. **A) Amino acids sequence of CALB**. Asp and Glu marked in yellow. Trp marked in blue. Sequence was obtained as reported.[2] B) **Amino acids sequence of TLL**. Asp and Glu marked in yellow. His in green. Trp in blue. Sequence was obtained as reported.[3]

Table S1. Amount of copper in each hybrid determined by ICP-OES.

Cu-enzyme nanohybrid	*Amount of Cu by ICP-OES (%)* [a]
**Cu-CALB**	35
**Cu-CAT**	34
**Cu-TLL**	44
**Cu-BTL**	37

[a]The measurement was performed of the solid material. 10 mg of the solid powder was treated with 5 mL of HCl (37% v/v) for digestion. Then, it was added with 5 mL of water, centrifuged and the clear solution analyzed by Cu content.

Table S2. Catechol activity of different enzymes against L-DOPA.

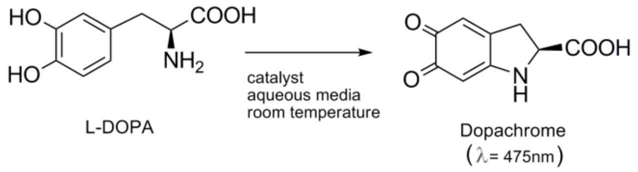

Entry	Sample	Solvent (pH)[a]	ΔABS/min
**2**	**CAT**	Distilled water	0
**3**	**CALB**	Distilled water	0
**4**	**TLL**	Distilled water	0
**5**	**BTL**	Distilled water	0
**6**	**TYR**	NaOAc (4)	0.006
**7**	**TYR**	Distilled water	0.274

[a] Conditions: 1 mM L-DOPA in 2 mL of 100 mM NaOAc at pH 4 or Distilled water. 50µL of an enzyme solution 1mg/mL was added

Table S3. Catechol activity of different enzymes against L-DOPAME.

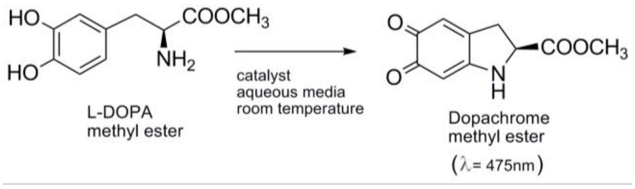

Entry	Sample	Solvent (pH)[a]	ΔABS/min
**2**	**CAT**	Distilled water	0
**3**	**CALB**	Distilled water	0
**4**	**TLL**	Distilled water	0
**5**	**BTL**	Distilled water	0
**6**	**TYR**	NaOAc buffer (4)	0.034
**7**	**TYR**	Distilled water	0.193

[a] Conditions: 1 mM L-DOPA in 2 mL of 100 mM NaOAc at pH 4 or Distilled water. 50µL of enzyme solution 1mg/mL was added in each case

References

1. https://www.uniprot.org/uniprot/A0A254TZH3.
2. https://www.uniprot.org/uniprot/B6DAC2.
3. https://www.uniprot.org/uniprot/O59952 .
